# SnoRNAs from the filamentous fungus *Neurospora crassa*: structural, functional and evolutionary insights

**DOI:** 10.1186/1471-2164-10-515

**Published:** 2009-11-08

**Authors:** Na Liu, Zhen-Dong Xiao, Chun-Hong Yu, Peng Shao, Yin-Tong Liang, Dao-Gang Guan, Jian-Hua Yang, Chun-Long Chen, Liang-Hu Qu, Hui Zhou

**Affiliations:** 1 Key Laboratory of Gene Engineering of the Ministry of Education, State Key Laboratory for Biocontrol, Sun Yat-sen University, Guangzhou 510275, PR China; 2 Centre National de la Recherche Scientifique (CNRS), UPR 2167, CGM, Gif sur Yvette, 91198, France

## Abstract

**Background:**

SnoRNAs represent an excellent model for studying the structural and functional evolution of small non-coding RNAs involved in the post-transcriptional modification machinery for rRNAs and snRNAs in eukaryotic cells. Identification of snoRNAs from *Neurospora crassa*, an important model organism playing key roles in the development of modern genetics, biochemistry and molecular biology will provide insights into the evolution of snoRNA genes in the fungus kingdom.

**Results:**

Fifty five box C/D snoRNAs were identified and predicted to guide 71 2'-O-methylated sites including four sites on snRNAs and three sites on tRNAs. Additionally, twenty box H/ACA snoRNAs, which potentially guide 17 pseudouridylations on rRNAs, were also identified. Although not exhaustive, the study provides the first comprehensive list of two major families of snoRNAs from the filamentous fungus *N. crassa*. The independently transcribed strategy dominates in the expression of box H/ACA snoRNA genes, whereas most of the box C/D snoRNA genes are intron-encoded. This shows that different genomic organizations and expression modes have been adopted by the two major classes of snoRNA genes in *N. crassa *. Remarkably, five gene clusters represent an outstanding organization of box C/D snoRNA genes, which are well conserved among yeasts and multicellular fungi, implying their functional importance for the fungus cells. Interestingly, alternative splicing events were found in the expression of two polycistronic snoRNA gene hosts that resemble the UHG-like genes in mammals. Phylogenetic analysis further revealed that the extensive separation and recombination of two functional elements of snoRNA genes has occurred during fungus evolution.

**Conclusion:**

This is the first genome-wide analysis of the filamentous fungus *N. crassa *snoRNAs that aids in understanding the differences between unicellular fungi and multicellular fungi. As compared with two yeasts, a more complex pattern of methylation guided by box C/D snoRNAs in multicellular fungus than in unicellular yeasts was revealed, indicating the high diversity of post-transcriptional modification guided by snoRNAs in the fungus kingdom.

## Background

Eukaryotic rRNAs contain a large number of nucleotide modifications including 2'-O-methylation and pseudouridylation which are directed by box C/D snoRNAs and box H/ACA snoRNAs, respectively [1,2]. These modifications are usually found in the conserved core regions of rRNAs, and they play important roles in ribosome function [3]. SnoRNAs are among the most numerous and functionally diverse non-coding RNAs currently known [4,5], existing widely in eukaryotes including human [6-8], plants [9-11], yeasts [12-15] and protists [16-19], as well as in Archaea [20]. This indicates that they are ancient molecules that arose over 2-3 billion years ago [21]. In addition to guiding the posttranscriptional modifications of rRNAs in eukaryotes and Archaea, snoRNAs also interact with spliceosomal RNAs [22], tRNAs [23,24], SL RNAs in trypanosomes [25], and at least one brain-specific mRNA in mammals [26]. Recently, snoRNA U50 was found to be a candidate tumor suppressor gene in prostate cancer [27]. The existence of substantial numbers of orphan snoRNAs indicates that snoRNAs also participate in diverse biological processes that remain to be identified [4].

SnoRNAs exhibit canonical sequence motifs and structural features. Box C/D snoRNAs carry the conserved box C (RUGAUGA, where R can be any purine) and D (CUGA) motifs near their 5' and 3' termini, respectively. Additionally, the variants of the C and D boxes, designated C' and D' box, are usually present internally [28]. Box H/ACA snoRNAs possess a hairpin-hinge-hairpin-tail secondary structure and two conserved sequence motifs, box H and box ACA. The hinge region contains the H box (ANANNA) and the tail consists of the ACA box located 3 nt before the 3' end [29,30]. The snoRNAs exert their functions by base-pairing with their targets and recruit related proteins to the sites of modification [31]. Box C/D snoRNAs can form 10-21 basepairs (bp) with multiple cellular RNAs. The methylated nucleotide in the target RNA is usually positioned 5 nt upstream of the D or D' box of the snoRNAs, the so called "D/D'+5" rule [6]. In box H/ACA snoRNAs, two short antisense sequences in one or both of the two hairpin domains form 9-13 basepairs with rRNA sequences that flank the target uridine to be converted to pseudouridine. The pseudouridine is always located 14 to 16 nt upstream from the H box or the ACA box of the snoRNA [29,30]. These structural and functional features of box C/D and H/ACA snoRNAs provide the parameters for identifying snoRNAs and their function.

The genomic organization of snoRNA genes displays great diversity in different organisms. In vertebrates, almost all snoRNA genes are located in the introns of host genes, with a few exceptions, such as U3 which are independently transcribed [4]. In plants and trypanosomatids, snoRNA genes are present in polycistronic clusters which encode both C/D and H/ACA snoRNAs [9,17]. A particular genomic organization, the intronic gene cluster, was first found in rice and then in *Drosophila melanogaster *[32,33]. Moreover, a unique genomic organization (dicistronic tRNA-snoRNA genes) has been characterized exclusively in plants [34]. The genomic organization of snoRNA genes differs largely in fungi. In the budding yeast *Saccharomyces cerevisiae*, apart from seven intronic snoRNA genes, the majority of snoRNA are encoded by independent genes as well as five polycistronic snoRNA gene clusters [12]. In contrast, most box C/D snoRNA genes from the fission yeast *Schizosaccharomyces pombe *are intron-encoded. This raises the question about the genomic organization and expression modes of snoRNA genes in the fungus kingdom. It is well known that multicellular fungi dominate the population of fungi. However, little is known about snoRNAs in multicellular fungi. It was thus of interest to determine the snoRNA genes from a multicellular fungi to shed light on these characteristics.

*Neurospora crassa *is a filamentous fungus sharing key components with animal cells in cellular physiology and genetics, contributing to the fundamental understanding of the genome defense system, DNA methylation, post-transcriptional gene silencing, cellular differentiation and development [35]. As a model eukaryote, the genome of *N. crassa *has been completely sequenced [36]. However, only four box C/D snoRNAs, snR39, snR52, snR60, snR61 (Rfam) were annotated in *N. crassa *. Recently, we identified three U3 snoRNA genes from *N. crassa*; each of them is independently transcribed and contains a small intron [37](Table 1). It is evident that the majority of the *N. crassa *snoRNAs remain to be identified. Meanwhile, a comparative genome analysis between yeast and multicellular fungi will provide insights into the evolution of snoRNA genes in the fungus kingdom. In this study, by combining computational and experimental methods, an extensive analysis of snoRNA genes from *N. crassa *was performed. Here, we present the first comprehensive list of two major families of snoRNAs from *N. crassa *, and further discuss the characteristics and evolutionary significance of the snoRNA genes.

**Table 1:** Box C/D snoRNAs identified in *N. crassa*

							Homologs				
**Name**^**a**^	**Len**^**b**^	**Chr**^**c**^	**Exp**^**d**^	Target site(s)	**Match**^**e**^	**G p**^**f**^	*S. p*	*S. c*	*A. t*	*H. s*	**Location**^**g**^
Nc CD1	125	III	C, N	26S-Am2242	10/0	D'	-	snR13	-	-	Intron
Nc CD2	99	V	C	26S-Um2379	13/0	D'	snR66	snR66	-	-	IR
Nc CD3	90	I	C, N	26S-Um2840	14/0	D'	-	-	snoR29		
Nc CD4	81	V	C, N	18S-Cm49	13/0	D'	-	-	-	-	Intron
Nc CD5	80	VI	C	26S-Am856	10/0	D	snR60-I	snR72	snoR72Y	-	Intron
				26S-Um2383	12/0	D'	snR78	snR78	snoR37	U52	
Nc CD6	76	I	C	26S-Um2687	11/0	D'	snR51-I	snR51	-	U41	Exon
Nc CD7	84	VI	C	26S-Gm2250	12/0	D'	snR75	snR75	U15	U15	Intron
Nc CD8	85	I	C	26S-Gm2751	13/0	D'	snR48	snR48	-	U60	IR
Nc CD9	81	I	C	18S-Gm1122	14/0	D	snR41-II	snR41	-	-	Intron
Nc CD10	104	I	C, N	26S-Um1039	12/0	D	-	-	-	-	Intron
				26S-Am3264	13/0	D'	-	-	-	-	
Nc CD11	79	I	C	18S-Am793	13/0	D'	snR53	snR53	snoR53Y	-	Intron
				U6-Am47	11/0	D'	snR53	-	-	mgU6-47	
				26S-Am356	15/0	D	-	-	-	-	
Nc CD12	85	VI	C	26S-Cm2159	10/0	D'	-	snR76	Ath119b	HBII-180	Intron
Nc CD13	84	VI	C	18S-Am538	12/0	D	snR41-I	snR41	snR41Y	U62A/B	Intron
Nc CD14	75	IV	C	26S-Am2288	10/0	D'	-	-	U79	U79	Intron
Nc CD15	73	I	C, N	18S-Am159	11/0	D'	-	-	-	-	Intron
				tRNA^Thr^-Um114	12/1	D	-	-	-	-	
Nc CD16	122	II	C	26S-Gm2357	13/0	D'	snR81	snR190	-	HBII-99	Intron
				26S-Gm1907		D'	-	-	-	U50	
Nc CD17	73	I	C	18S-Am154	13/0	D	-	-	-	U45A/C	Intron
				26S-Gm2875	12/0	D'	-	-	snoR34	HBII-210	
Nc CD18	88	V	C, N	18S-Cm1004	11/0	D	snR79	snR79	-	-	Intron
Nc CD19	75	III	C	18S-Gm1423	13/0	D'	snR56	snR56	snoR19	U25	Intron
Nc CD20	75	VI	C, N	26S-Um2372	13/0	D	snR88	-	snoR58	-	Intron
Nc CD21	69	VI	C	18S-Am28	13/0	D'	snR74	snR74	U27	U27	Intron
Nc CD22	79	I	C	26S-Um1866	14/0	D'	snR62	snR62	U34	U34	Intron
				18S-Um893	11/0	D	-	-	-	-	
Nc CD23	72	V	C, N	26S-Gm2773	11/0	D'	snR38	snR38	snoR38Y	snR38	Intron
Nc CD24	75	VI	C	18S-Um575	13/0	D'	snR77	snR77	snoR77Y	HBII-135	Intron
				U5-Am62	15/0	D	-	-	-	-	
Nc CD25	81	I	C, N	26S-Am846	13/0	D'	-	-	-	-	Intron
Nc CD26	77	I	C	U2-Gm183	11/0	D'	-	-	-	-	Intron
Nc CD27	85	VI	C	26S-Am2904	15/0	D'	snR71	snR71	U29	U29	Intron
				26S-Cm2906	15/0	D'	snR69	snR69	snoR69Y	-	
Nc CD28	78	II	C	26S-Am1845	12/0	D'	-	-	snoR33	U95	Intron
				26S-Am1859	14/0	D	-	-	-	-	
Nc CD29	101	II	C	U2-Am31	16/0	D	-	-	-	SCARNA9	Intron
				5.8S-Am42	14/0	D'	-	-	snoR9	-	
Nc CD30	84	I	C, N	18S-Cm584	12/0	D'	-	-	-	-	IR
Nc CD31	101	VI	C, N	26S-Cm2917	13/0	D'	snR73	snR73	U35	U35	Intron
Nc CD32	82	VII	C	18S-Am161	11/0	D	-	-	snoR18	U44	Intron
				18S-Um167	11/0	D'	-	-	snoR122	U45A/B	
Nc CD33	102	III	C	26S-Am2218	13/0	D	snR63	snR63	U46	U46	Intron
Nc CD34	69	IV	C	18S-Um1265	12/0	D'	snR55	snR55	snoR34	U33	Intron
Nc CD36	90	I	C	26S-Cm2299	10/0	D'	snR64	snR64	snoR44	U74	Intron
Nc CD37	97	II	C	Cleavage			U14	U14	U14	U14	Intron
				18S-Cm411	15/0	D	U14	U14	U14	U14	
Nc CD38*	80	IV	C	26S-Am1114	12/0	D'	snR61	snR61	U38	U38	Intron
				26S-Am2858	10/0	D'	-	-	-	-	
Nc CD39	73	I	C	26S-Am897	13/0	D'	snR83	-	-	-	Intron
				26S-Am375	11/0	D	-	-	-	-	
Nc CD40	107	IV	C, N	26S-Am2062	11/0	D'	-	-	-	-	Intron
Nc CD41	72	II	C	18S-Gm1423	12/0	D'	snR56	snR56	snoR19	U25	Intron
				26S-Cm1489	12/0	D	-	-	U49	mgh28S-2409	
Nc CD42*	92	II	N, R	18S-Am417	12/0	D	snR52	snR52	-	U83	Intron
Nc CD43	96	VI	N, R	18S-Gm559	11/0	D'	snR80	-	-	-	Intron
Nc CD44	85	IV	N, R	18S-Um1227	15/0	D'	snR82	-	snoR14	HBII-55	Intron
				26S-Cm776	11/0	D	-	-	-	-	
Nc CD45	102	I	N, R	26S-Um3021	11/0	D	-	-	-	-	IR
Nc CD46A	89	VII	N, R	26S-Am635	11/0	D'	U18	U18	U18	U18	Intron
Nc CD46B	89	I	N, R	26S-Am635	12/0	D'	U18	U18	U18	U18	Intron
Nc CD47*	75	III	N, R	26S-Gm785	16/0	D	snR39b	snR39b	snR39BY	snR39b	Intron
Nc CD48*	91	V	N, R	26S-Am797	14/0	D'	snR60-I	snR60	U80	U80/U77	Intron
				26S-Gm888	17/0	D	snR60-II	snR60	U80	U80	
Nc CD49	91	I	N, R	26S-Um2682	12/0	D	-	snR67	-	-	Intron
				18S-Am971	13/0	D'	snR54	snR54	U59	U59A/B	
Nc CD50	98	I	N, R	26S-Cm1418	15/0	D'	U24	U24	U24	U24	IR
Nc CD51	87	VII	N, R	26S-Am1430	13/0	D'	U24b	U24	U24	U76	Intron
Nc CD52	177	VII	C, N	tRNA-Am43	11/0	D'	-	-	-	-	IR
				tRNA^Leu^-Am90	12/0	D'	-	-	-	-	
Nc CD53	212	IV	C, N	Orphan			-	-	-	-	IR
Nc CD54	125	V	C, N	26S-Um667	10/0	D	-	-	-	-	IR
Nc CD55	137	IV	C, N	Orphan			-	-	-	-	Intron
Nc U3A	262	I	C, N	Cleavage			-	-	-	-	RE
Nc U3A-2	184	I	C, N				-	-	-	-	RE
Nc U3A-3	75	I	C, N				-	-	-	-	RE
Nc U3B	270	I	C, N	Cleavage			-	-	-	-	RE
Nc U3B-2	191	I	C, N				-	-	-	-	RE
Nc U3C	275	II	-	Cleavage			-	-	-	-	RE

^a ^The box C/D snoRNAs were numbered according to the order of identification. ^b ^Len, cDNA length of the snoRNA. ^c ^Chr, chromosomal location of snoRNA gene. ^d ^Exp, expression situation. C, N, R, snoRNA was identified by cDNA library, northern blotting analysis, and reverse transcription analysis, respectively. ^e ^target match, (Watson-crick pairs+G*U)/mismatch. ^f ^Gp, guide position. ^g ^IR, Intergenic region; RE, Repeat element. The genes marked with asterisks indicate that the genes were annotated in the *Neurospora crassa *database but were not detected by experimental methods. The data for *S. p *snoRNAs were cited from Luo (2004) [38] and Bi *et al*. (2007) [39]. *A. t *snoRNAs and modifications are from the plant database http://bioinf.scri.sari.ac.uk/cgi-bin/plant_snorna/conservation. *S. c *snoRNAs and modifications are from the yeast snoRNA database at UMass-Amberst http://people.biochem.umass.edu/fournierlab/snornadb/main.php. Abbreviation: *S. p*, *S. pombe*; *S. c*, *S. cerevisia*; *A. t*, *A. thaliana*; *H. s*, *H. sapiens*.

## Results

### Identification of 55 box C/D and 20 box H/ACA snoRNAs from *N. crassa*

We initially carried out the genome-wide analysis of snoRNAs from *N. crassa *by employing the snoscan [12] and snoGPS programs [13]. From this database search, 89 box C/D and 131 box H/ACA snoRNA candidates were predicted (see Methods). To validate the snoRNA candidates and identify more novel snoRNAs from *N. crassa *, the box C/D and box H/ACA snoRNA-specific library of *N. crassa *were respectively constructed from mixed-stage mycelium and spores using anchored primers (18, and see Methods). To exclude the highly abundant clones and enrich the novel RNA species in our analysis, the radiolabelled oligonucleotides were used to screen the cDNA libraries (~1800 clones in the box C/D and ~ 4000 clones in box H/ACA snoRNA libraries). Subsequently, a total of 338 and 278 clones from box C/D and box H/ACA snoRNA libraries were sequenced, respectively. Taken together, 65 snoRNAs including 45 box C/D (Table 1) and 20 box H/ACA snoRNAs (Table 2) were identified. Twenty eight box C/D snoRNAs from the cDNA library were covered by the snoscan results. However, only three H/ACA snoRNAs overlapped with snoGPS results. Because the data from the computational search of H/ACA snoRNAs may include excessive false-positive candidates, they were not included for further analyses in this study.

**Table 2:** Box H/ACA snoRNA genes in *N. crassa*

						Homologs				
**Name**^**a**^	**Len**^**b**^	**Chr**^**c**^	**Exp**^**d**^	Target site (s)	**G p**^**e**^	*S.p*	*S.c*	*A.t*	*H.s*	**Location**^**f**^
Nc ACA1	136	III	N, R	18S-Ψ105	H	-	snR44	-	ACA36	Intron
				26S-Ψ1037	ACA	-	snR44	-	-	
Nc ACA2	159	III	N, R	26S-Ψ1868	H	-	-	-	-	IR
				26S-Ψ2313	ACA	-	snR82	-	-	
Nc ACA3	217	V	C, N	26S-Ψ401	H	-	-	-	-	IR
				26S-Ψ2095	ACA	-	snR3	-	ACA6	
Nc ACA4	206	II	C, N	18S-Ψ463	H	-	snR189	-	-	IR
Nc ACA5	187	I	C, N	18S-Ψ996	ACA	-	snR31	snoR5	ACA8	IR
Nc ACA6	165	VII	C, N	26S-Ψ940	H	-	snR8	-	ACA56	IR
				26S-Ψ1105	ACA	snR5	snR5	-	-	
Nc ACA7	167	V	C, N	Orphan	ACA	-	-	-	-	IR
Nc ACA8	192	II	C, N	18S-Ψ1509	ACA	-	-	-	-	IR
Nc ACA9	201	V	C, N	Orphan		-	-	-	-	Intron
Nc ACA10	309	II	C, N	Orphan		-	-	-	-	3'UTR
Nc ACA11	176	I	C, N	Orphan		-	-	-	-	IR
Nc ACA12	296	II	C, N	Orphan		-	-	-	-	IR
Nc ACA13	208	I	C, N	Orphan		-	-	-	-	IR
Nc ACA14	234	II	C, N	26S-Ψ984	ACA	snR5	snR5	snoR81	ACA52	IR
Nc ACA15	233	II	C, N	26S-Ψ2902	H	-	snR37	-	ACA10	IR
Nc ACA16	189	II	C, N	Orphan		-	-	-	-	Exon+3'UTR
Nc ACA17	189	II	C, N	18S-Ψ1733	H	-	-	snoR88	-	IR
Nc ACA18	186	V	C, N	26S-Ψ2309	H	-	-	-	E2	IR
Nc ACA19	178	V	C, N	26S-Ψ1666	ACA	-	-	-	-	IR
Nc ACA20	160	V	N, R	26S-Ψ2228	ACA	-	snR84	-	Undet	IR

^a ^All the box H/ACA snoRNAs were numbered according to the order of identification. ^b ^Len, cDNA length of the snoRNA. ^c ^Chr, chromosomal location of snoRNA gene. ^d ^Exp, expression situation. C, N, R, snoRNA was identified by cDNA library, northern blotting analysis, and reverse transcription analysis, respectively. ^e ^Gp, guide position. ^g ^IR, Intergenic region. The data for *S. p *snoRNAs were cited from Luo (2004) [38]. "Undet" indicates that the snoRNA has not been identified in the human genome although the corresponding modification site was detected. Abbreviation: *S. p*, *S. pombe*; *S. c*, *S. cerevisia*; *A. t*, *A. thaliana*; *H. s*, *H. sapiens*.

**Figure 1:**
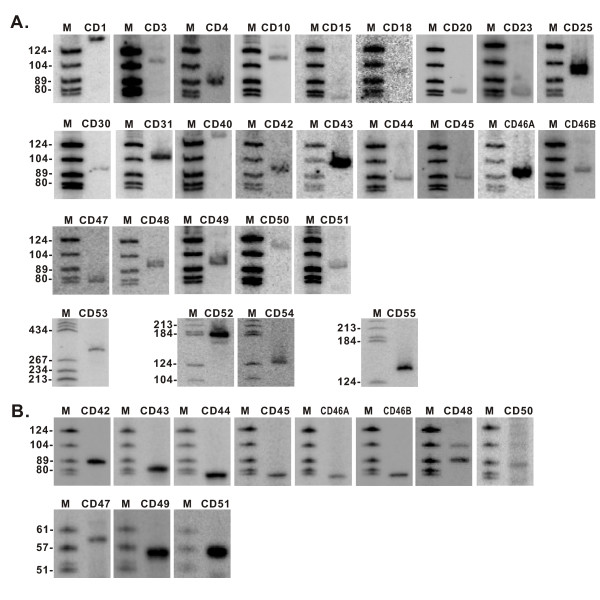
**Northern blot and RT analyses of box C/D snoRNAs from N. crassa**. A. Northern blot analyses of box C/D snoRNAs. B. Reverse transcription analyses of box C/D snoRNAs generated from the computational screen. Lane M, molecular weight marker (pBR322 digested with Hae III and 5'-end -labeled with [γ-^32^P]ATP).

**Figure 2:**
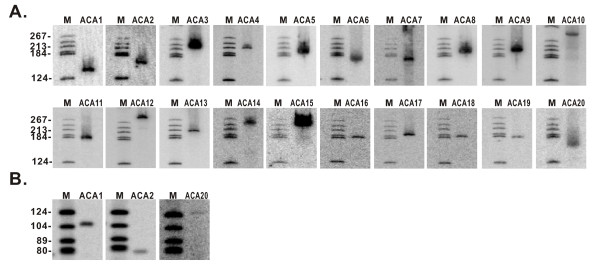
**Northern blot and RT analyses of box H/ACA snoRNAs from N. crassa**. A. Northern blot analyses of box H/ACA snoRNAs. B. Reverse transcription analyses of the three box H/ACA snoRNAs overlaps with the computational screen. Lane M, molecular weight marker (pBR322 digested with Hae III and 5'-end -labeled with [γ-^32^P]ATP.

The snoRNA candidates identified by cDNA cloning or the snoscan program were subsequently confirmed by northern blot and/or reverse transcription analyses. The expression of 27 box C/D and all 20 box H/ACA snoRNAs were positively detected as expected (Figure 1 and 2). Among these snoRNAs, the sequence of CD31 snoRNA obtained from the cDNA cloning appears uncompleted; it corresponds to the second half of CD31 full-length which is further validated by the northern blotting.

Together, through the combination of computational analysis and construction of the specialized cDNA libraries, 55 box C/D and 20 box H/ACA snoRNAs were identified and all the snoRNAs are denominated according to the order of identification (Table 1 and 2).

In most cases (86%) the C and D boxes in snoRNAs are highly conserved when compared to the consensus sequence (UGAUGA and CUGA, see Additional file 1). However, the C' and D' box are nonconserved and exhibit much more sequence-degeneracy than their C and D box counterparts. In some instances, the C' and D' box cannot be unambiguously inferred as a result of the absence of the functional region. Generally, the box C/D snoRNAs from *N. crassa *are similar to their metazoan and yeast counterparts in size. However, the sizes of box H/ACA snoRNAs from *N. crassa *are peculiar. Almost all of them are larger than 160 nt (Figure 2), reminiscent of some irregular box H/ACA snoRNAs in *S. cerevisiae*. These observations show that the *N. crassa *snoRNAs have their own unique sequence and structural features (see Additional file 2 and 3).

### Functional properties of the *N. crassa *box C/D and box H/ACA snoRNAs

Based on the modification rules of snoRNAs [2] , 55 box C/D snoRNAs from *N. crassa *were predicted to direct 71 methylations. These include 64 methylations on rRNAs which comprise 43 methyls on 26S rRNA, 20 methyls on 18S rRNA and one methyl on 5.8S rRNA (see Additional file 4A). The remnant seven methylations consist of four methyls on snRNAs and three methyls on tRNAs (see Additional file 4B and 4C). Furthermore, the structure and function elements of U14 which participate in the processing of pre-rRNA were unambiguously identified. Interestingly, two different methylated sites were predicted to be guided by the same functional element of a single snoRNA CD27. Two box C/D snoRNAs (CD53 and CD55) lack the sequences complementary to either rRNAs or snRNAs and therefore belong to orphan snoRNAs. Fourteen box H/ACA snoRNAs were predicted to guide 17 pseudouridine sites of rRNAs (see Additional file 5), and no pseudouridine sites on snRNAs were predicted. The remaining six box H/ACA snoRNAs were also classified into an orphan snoRNA family lacking functional region complementary to rRNA, tRNA or snRNA. A different modification pattern appears in *N. crassa *as compared to the two yeasts *S. cerevisiae*, and *S. pombe *(see discussion)[38,39].

**Figure 3:**
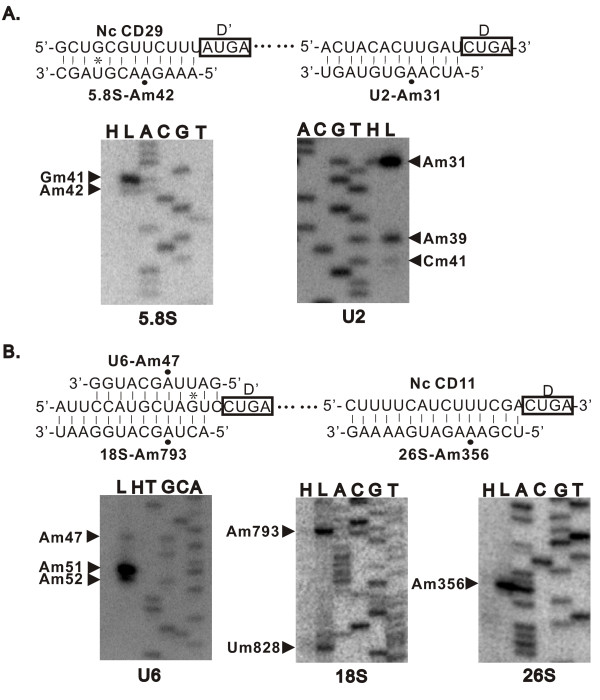
**Base-pairing model and verification of modification guided by CD11 (A) and CD29 (B)**. Black dots indicate nucleotides predicted to be methylated. Lane H, control reaction at 1.0 mM dNTP; Lane L, primer extension at 0.004 mM dNTP, and A, C, G and T lanes, the rDNA sequence ladder. Black triangles indicate potential methylation sites.

Interestingly, a novel snoRNA, CD29, possesses two guide elements that can form duplexes with U2 snRNA and 5.8S rRNA for 2'-O-methylation. Primer extension mapping of 2'-O-methylated nucleotides of the U2 snRNA and 5.8S rRNA in the presence of low concentration of dNTPs resulted in stop signals at the G32 and A43 residues, indicating that U2-A31 and 5.8S-A42 are methylated (Figure 3). We have identified cognates of CD29 in other filamentous fungi, however, these cognates only possess the guide sequence for the methylation of U2 snRNA. This suggests that CD29 evolves from the snoRNA with a single guide function. This is reminiscent of the human small Cajal body-specific RNAs (scaRNAs) that can guide modification of the RNA polymerase II-transcribed snRNAs such as U2 snRNA. The comparative analyses revealed that CD29 and its homologs in fungi have one functional element similar to that of human SCARNA9 which was first known as Z32 (GeneBank accession no. AJ009638), and therefore was homologous to this human scaRNA. In addition, we characterized a multi-function box C/D snoRNA, CD11, in *N. crassa *. CD11 has the potential to direct a methylation in U6 snRNA, and two methylations in 18S and 26S rRNAs, respectively (Figure 3). Interestingly, the CD11 is also partially similar to mgU6-47 in mammals [40], but possesses a novel function that can guide a *N. crassa*-specific methylation on 26S rRNA at A356.

### Genomic organization and expression of the snoRNAs in *N. crassa*

**Figure 5:**
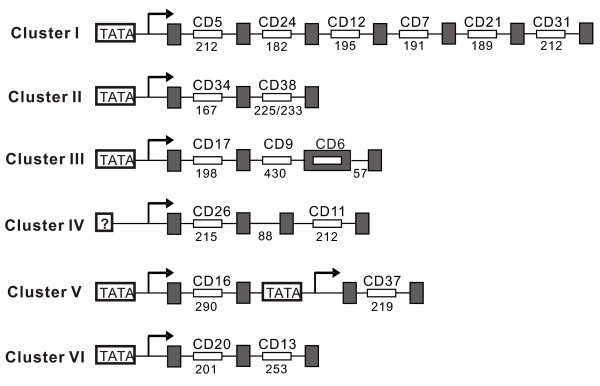
**Schematic representation of snoRNA gene clusters in N. crassa**. The open and gray boxes represent snoRNAs and exons, respectively. The number below indicates the length (in nucleotides) of introns. Thinner lines indicate introns. Note: figure not drawn to scale.

The genomic organization of the snoRNA genes in *N. crassa *exhibits great diversity. Among the 55 box C/D snoRNAs, forty five snoRNA genes are intron-encoded in protein-coding or non-coding host genes. The remaining nine were found in the intergenic regions with a putative polymerase II promoter upstream and appeared independently transcribed. Meanwhile, six gene clusters that only encode box C/D snoRNAs were identified from *N. crassa *. Interestingly, an exon-encoded snoRNA (CD6) was identified in the snoRNA gene cluster III in contrast to another two intron-encoded snoRNAs (CD9 and CD17) in the same cluster (Figure 5). Of 20 box H/ACA snoRNA genes, 16 are located in intergenic regions and two are intron-encoded. In particular, two snoRNA genes (ACA10 and ACA16) are located in the 3' UTR of two hypothetical protein genes, one of which is similar to phosphoglycerate mutase. Obviously, different strategies dominate in the expression of the two families of snoRNA genes in *N. crassa *.

**Figure 4:**
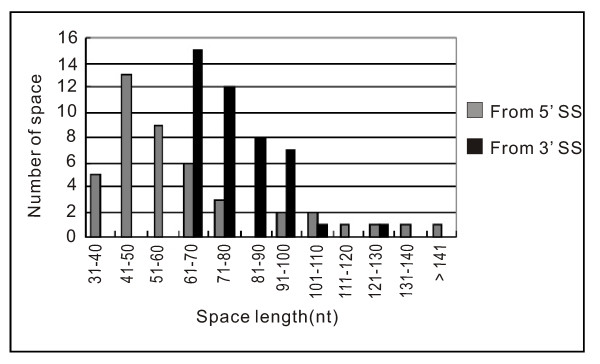
**The distance distributions from the intronic snoRNA genes to the 3' and 5' splicing sites of host gene introns**. The gray and black bars represent space lengths from the 3' and 5' splicing sites (SS), respectively, to the snoRNA genes.

In accordance with the mode of one snoRNA per intron in vertebrates [4], a large proportion of the box C/D snoRNA genes (45 of 55) are located within introns of the host genes. The distances from the intronic snoRNA genes to the 3' splice sites of introns, which has been proven to be important for the effective processing of intronic snoRNAs from their host mRNA precursors [41,42], resemble those in *D. melanogaster *[32,41,42]. The distances from the snoRNA genes to the 5' splice sites appear to mainly be between 41 to 60 nt, similar to those in human[41] (Figure 4).

Remarkably, five (cluster I to V) of the six box C/D snoRNA gene clusters arehighly conserved between yeast and *N. crassa *(Figure 5). Although these host genes were not well annotated for their introns and exons in the *N. crassa *genome, canonical intron splicing sequences were observed flanking every cluster of snoRNA genes. To further confirm this observation, the mature RNA transcripts were identified with the expected sizes by cloning and sequencing of RT-PCR products. It is worth noting that two snoRNA genes, CD16 and CD37, in the cluster V are validated to be co-transcribed by RT-PCR and sequencing, though each of the snoRNA genes in the cluster has a putative promoter upstream. Intriguingly, the putative promoter upstream of CD37, a homologue of U14, would play a role in guaranteeing and promoting the function of U14 that has been demonstrated vital in diverse eukaryotes. Our results further revealed that the genomic organization of the host genes for these five clusters is most like the UHG gene in animals. The host genes of Cluster I to V only contain short open reading frames with length ranging from 159 bp to 267 bp, suggesting the little potential for protein coding just like the *gas 5 *[43].

**Figure 6:**
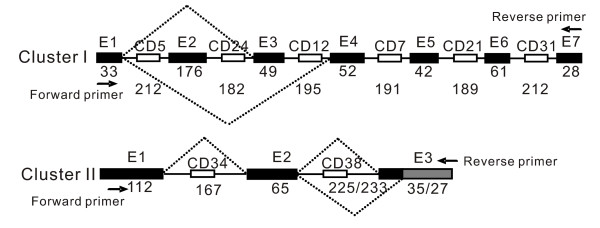
**Alternative splicing in the expression of snoRNA gene cluster I and II in N. crassa**. The open and black boxes represent snoRNAs and exons, respectively. The number below indicates the length (in nucleotides) of exons and introns. Thinner lines indicate introns and dashed lines indicate splicing activities. Arrows indicate the primers used in RT-PCR analysis.

Unexpectedly, various alternative splicing events were found in the processing of polycistronic transcripts from the snoRNA gene clusters I and II by analyzing cDNA sequences from RT-PCR of the transcripts (Figure 6). In cluster I, two alternatively spliced transcripts, differing by the absence of exon 2 or exon 2 plus exon 3 were detected. The pattern of alternative splicing in the expression of cluster II was contingent on an alternative 3' splice site that allows the lengthening or shortening of exon 3.

## Discussion

### High diversity of post-transcriptional modification predicted by snoRNAs in fungi

**Figure 7:**
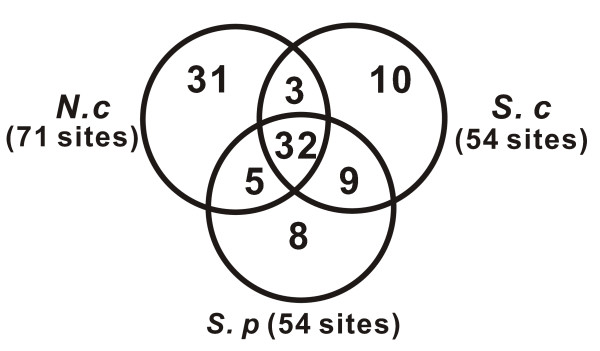
**Venn diagram of the relationship of methylations in three fungi**. The number of each part of the methylations is shown. Abbreviation: *N.c*, *N. crassa*; *S. c*, *S. cerevisiae*; *S. p*, *S. pombe*.

Identification of guide snoRNAs in diverse organisms can provide valuable information towards understanding RNA modification patterns and their function [18]. It is interesting to compare the pattern of modifications on target RNAs of *N. crassa *to those described in the two yeasts, *S. cerevisiae *and *S. pombe*. Among 71 methylations predicted by the guide snoRNAs in *N. crassa *, 32 represent the most highly conserved modifications shared by the multicellular fungi and the yeasts, and 31 (43.7%) are modifications that have not yet been reported in other fungi when compared with the two unicellular yeasts(Figure 7). In the yeasts, only ten and eight methylations are *S. cerevisiae*-specific and *S. pombe*-specific, respectively. Our results imply a more complex modification pattern in multicellular fungi than in unicellular yeasts. They also reveal the high diversity of post-transcriptional modification of RNAs in the fungus kingdom as it has been shown that about 40% of methylations are species-specific in a protozoan *Trypanosoma *[17]. The species-specific modifications highlight the different modification patterns and their peculiar importance. Although eliminating a single modification does not have a dramatic effect on the ribosome [44], loss of three to five modifications in an intersubunit bridge of the ribosome (helix 69) impairs growth and causes broad defects in ribosome biogenesis and activity [45]. On the other hand, early studies have demonstrated that ribosome modifications play roles in determining antibiotic resistance or sensitivity [15,46]. Thus the species-specific modifications have potential use in finding therapeutic targets for prevention and treatment of diseases caused by some eukaryotic pathogens.

Another interesting observation in this study was the presence of duplexes between box C/D snoRNAs and tRNAs (tRNA^Trp ^and tRNA^Leu ^from *N. crassa *Database). Duplexes between tRNA and snoRNAs have been also found in *C. elegans *[24] and recently in *Plasmodium falciparum *[47]. tRNA modification guided by snoRNAs has been also reported in Archaea [23]. This study provides for the first time a prediction of fungal snoRNAs and their potential target sites in tRNAs, although these remain to be confirmed by further experiments.

### Structural and functional evolution of snoRNAs in fungi

**Figure 8:**
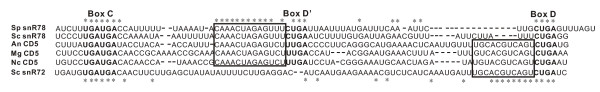
**Alignment of homologous snoRNAs from three multicellular fungi and two yeasts**. Conserved box elements are bold and boxed regions denote antisense elements. Stars indicate conserved nucleotides. Sp, *S. pombe*; Sc, *S. cerevisiae*; An, *Aspergillus niger*; Mg, *Magnaporthe grisea*; Nc, *N. crassa *.

Our study demonstrates the extensive separation and recombination of functional regions occurring during the evolution of snoRNA genes in fungi. For instance, the CD5 snoRNA in *N. crassa *possesses two conserved guiding elements. In *S. cerevisiae*, however, the conserved function of CD5 is executed by two independent snoRNAs, snR72 and snR78, with a single functional element [48] (Figure 8). This suggests that CD5 may have evolved as a double-guide snoRNA through recombination of two different halves of two ancestral single-guide snoRNAs. The other possibility is that a gene duplication of a double-guide snoRNA gene in *S. cerevisiae *led to specialization of each paralog to only target one modification site followed by loss of the other guide element for both paralogs. Another example is CD50 and CD51 that carry a conserved guiding function for U24 and U24b in *S. pombe*, respectively. In contrast, the U24 in *S. cerevisiae *has two guiding functions. Comparative analyses revealed that the structure and function of U24 are well conserved among the budding yeast and the flowering plants *A. thaliana *and rice, but the homologues of the *S. cerevisiae *U24 exist as two independent snoRNAs in other distant eukaryotes, such as human and mouse [49]. This suggests that U24 snoRNA gene has evolved in two pathways, with one leading to a dual functional snoRNA gene and the other separating the guiding functions and giving rise to two independent snoRNA genes.

**Figure 9:**
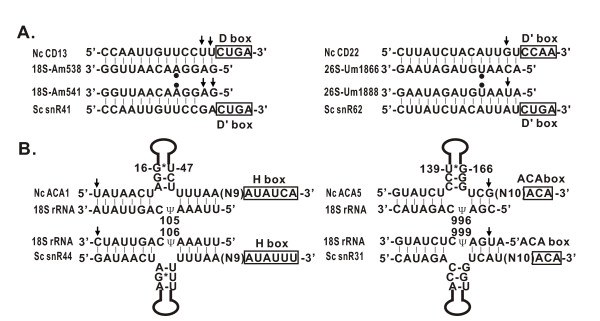
**Coevolution between snoRNAs and their targets**. A. Nucleotides of box C/D snoRNAs in the complementary region were changed in coordination with its target rRNA maintaining phylogenetical conservation of rRNA methylated sites. B. Nucleotides changed in box H/ACA snoRNAs respond to specific changes in the 18S rRNA of *N. crassa *. The nucleotides marked by black dot represent the 2'-O-methylation. Basepairs changed are indicated by arrows.

It has been demonstrated the reciprocal evolutionary change between snoRNA complementary region and their rRNA target sequence in plants and nematodes[9,24]. Our analyses indicate that co-evolution between snoRNAs and rRNAs exists widely in *N. crassa *(Figure 9) and plays an important role in preservation of phylogenetic conserved methylated sites of rRNAs which is essential for protein synthesis.

### RIP may impact on the generation of snoRNA isoforms by gene duplication and transposition

SnoRNA gene isoforms or variants exist widely in diverse organisms, particularly in plants. For example, 97 box C/D snoRNAs with a total of 175 different gene variants were identified in the *A. thaliana *genome [50], and 346 gene variants encoding 120 box C/D snoRNAs were found in *Oryza Sativa *[9]. Compared with the plant snoRNAs, only a paucity of yeast snoRNA paralogs was detected because of a relatively small compact genome (~12 Mb for *S. cerevisiae*). The *N. crassa *genome (~ 40 Mb) is three-fold larger than that of the yeast; however, most snoRNA genes in this species are singleton. Why are the snoRNA genes devoid of isoforms in the *N. crassa *genome? It is known that a mutagenic process termed repeat-induced point mutation (RIP) has a profound impact on *N. crassa *genome evolution, which has greatly slowed the creation of new genes through genomic duplication and resulted in a genome with an unusually low proportion of closely related genes [51]. Of the predicted 10082 protein-coding genes, only six pairs (12 genes) share >80% nucleotide or amino-acid identities in their coding sequences [36]. RIP identifies duplications that are greater than ~400 bp (~1 kb in the case of unlinked duplications) and induces C:G to T:A during the sexual cycle [52,53]. Early studies have provided clear evidence of retrotransposons inactivated by RIP [54,55]. The analysis of the *N. crassa *genome sequence also revealed a complete absence of intact mobile elements [36]. Therefore the creation of new genes including snoRNA genes or their host genes through gene duplication and transposition seems to be impeded. It has been proposed that most, if not all paralogs in *N. crassa *duplicated and diverged before the emergence of RIP [51]. We have identified three U3 snoRNA gene variants, NcU3A, NcU3A-2 and NcU3A-3 in *N. crassa *(37). The sequence analysis revealed that these molecules have undergone nucleotide substitutions rather than RIP according to the calculation method previously reported [36]. In the case of CD46A and CD46B, we speculate that the two snoRNA gene isoforms may have duplicated and diverged before the emergence of RIP.

### Alternative splicing in the expression of non-coding RNA genes with introns

It is well known that alternative splicing is an important and widespread process where one gene produces more than one type of mRNA which is then translated into different proteins in multicellular organisms [56]. Bioinformatic analysis indicates that 35-65% of human genes are involved in alternative splicing, which contributes significantly to human proteome complexity [57,58]. However, alternative splicing is rarely reported for non-coding RNA genes which encode multiple introns. In this study, we identified several alternative splicing events that occurred in the processing of RNA precursors transcribed from the snoRNA gene cluster I and II of *N. crassa*. It has been reported that the mouse *gas5 *gene, a non-coding RNA and snoRNA host gene, had four alternative splicing transcripts [43]. Although different in snoRNA composition, the snoRNA gene clusters in *N. crassa *are most like UHG genes resembling *gas5*. Our results show that alternative splicing occurs frequently in the expression of snoRNA host genes in lower eukaryotes. This lends support to the concept that alternative splicing may be an ancient mechanism in regulating the expression of both protein-coding and non-coding RNA genes with introns. More work is necessary to elucidate the biological significance of the alternative splicing in the expression of non-coding RNA genes.

## Conclusion

In this study, we report the first extensive identification of box C/D and box H/ACA snoRNAs from the filamentous fungus *N. crassa *using a combination of computational and experimental method. The repertoire characteristics, targets, genomic organization and the unique function of the *N. crassa *snoRNA genes were extensively compared with those of potential orthologues in close and distant organisms such as *S. pombe*, *S. cerevisiae, A. niger*, *M. grisea*, *A. thaliana *and *H. sapiens *. Our results improve annotation of snoRNA genes in the *N. crassa *genome, an important model filamentous fungus, and provide insights into the characteristics and evolutionary significance of the snoRNA genes in the fungus kingdom.

## Methods

### Strains and Media

The *N. crassa *wild-type strain (As 3.1604, purchased from the China General Microbiological Culture Collection Center) was used for the construction of the cDNA library and all RNA analyses. The strain was grown in PSA medium (2% sucrose, 20% extract of potato) at 30°C. The *Escherichia coli *strain TG1 grown in 2YT (1.6% Bacto tryptone, 1% yeast extract, 0.5% NaCl) liquid or solid medium was used for cloning procedures.

### Construction and screening of cDNA library

We prepared total RNA from *N. crassa *culture according to the guanidine thiocyanate-phenol-chloroform procedure described by Chomoczynski *et al *[59]. Small RNA (~20 μg) was fractionated by 50% PEG-8000 and 0.5 M NaCl. The construction of cDNA library were performed as described previously with minor modifications (see Additional file 6) [60]. After randomly sequencing clones, we employed dot hybridization to screen the colony PCR products with P47 and P48 as described by Liu *et al*. [37] We sequenced clones exhibiting the lowest hybridization signal.

### Computational identification of box C/D snoRNA genes

Genomic sequences of *N. crassa *[36] available at http://www.broad.mit.edu/annotation/genome/neurospora/Home.html (*N. crassa *assembly 7) were downloaded and searched for potential box C/D snoRNAs target rRNA/snRNA using snoscan [12] with default parameters. Methylated sites prepared for the snoscan included the conserved methylated nucleotides of *S. cerevisiae *(yeast snoRNA database), *H. sapiens *(snoRNA-LBME-db), and *D. melanogaster *[32]. The snoscan results were processed by an in-house developed perl program for candidate selection. A sequence with the following characteristics was considered as candidate: ① box C motif bit score ≥ 7.48, box D motif bit score ≥ 8.05, ② the guide bit score ≥ 18.65, the guide sequence and the target sequence can form a concatenated 10 bp duplex with at most 1 GU pair allowed, or can form a concatenated 9 bp duplex with high GC content. ③ if the guide region is adjacent to the D' box, the length of spaces between box C and guide sequence must be ≤ 20 bp. If the guide region is adjacent to the D box, the length of spaces between box C and guide sequence must be between 40 and 85 bp. ④ total sequence length between 75 bp and 130 bp, total overall bit score ≥ 20. The candidates within CDS region predicted by Broad/Whitehead Institute automatic gene calling software (a combination of manual annotation, FGENESH, GENEID, and GENEWISE) [36] were removed. The BLAST program [61] was used to search gene variants of all novel snoRNA genes to establish the snoRNA gene isoforms. About 1 kb of flanking sequences of the snoRNA gene candidates was searched further for possible box C/D snoRNA genes and additional non-canonical C/D gene candidates.

### Northern blot analysis

An aliquot of 30 μg total RNA was separated by electrophoresis on an 8% polyacrylamide gel containing 8 M urea and electrotransferred onto nylon membrane (Hybond-N+; Amersham) using semi-dry blotting apparatus (BioRad). After immobilizing RNA using a UV cross-linker, northern blot hybridization was performed as previously described [49].

### Reverse transcription and mapping of ribose methylation

Reverse transcription was carried out in a 20 μl reaction mixture containing 15 μg of total RNA and a corresponding 5'-end-labeled primer. After denaturation at 65°C for 5 min and then cooling to 42°C, 200 units of M-MLV reverse transcriptase (Promega) were added and extension carried out at 42°C for 1 hour. The cDNA was separated on an 8% polyacrylamide gel (8 M urea) and then analyzed with an imager.

The mapping of rRNA methylated sites was determined by primer extension at low dNTP concentrations as described previously [40,62]. Briefly, the *N. crassa *18S and 26S rDNA were amplified by PCR with the primer pair Nc18SF/Nc18SR and Nc26SF/Nc26SR, respectively, and then cloned into the pMD-18T vector (Takara). The plasmid DNA insert was directly sequenced with the same primer used for reverse transcription and run in parallel with the reverse transcription reaction on an 8% polyacrylamide gel (8 M urea).

### RT-PCR analysis

15 μg of total RNA was reverse transcribed with 200 U of M-MLV reverse transcriptase (Promega) using the box C/D snoRNA gene cluster specific reverse primers (see Additional file 7) in a 20 μl reaction mixture as described above for reverse transcription and mapping of ribose methylations. The negative RT control was carried out without M-MLV reverse transcriptase. We designed two specific antisense oligonucleotides: the first reverse primer used in the reverse transcription reaction overlaps the last several nucleotides of the second reverse primer used in the PCR reaction to help avoid non-specific PCR products. After 1 h at 42°C, 2 μl of RT reaction was used for PCR amplification with the second reverse primer and the corresponding forward primer (see Additional file 7) in a final volume of 20 μl. The positive PCR control was performed on *N. crassa *genomic DNA with the same pair of primers. Negative PCR control was performed on 2 μl of the negative control RT reaction with the same pair of primers. The PCR program: 30 cycles of denaturation (30 s, 94°C), annealing (30 s, 50-55°C), and extension (1-2 min, 72°C), following by a final extension (10 min, 72°C). The PCR product was purified from a 1.5% agarose gel with the QIAquick Gel extraction Kit (QIAGEN) and cloned into pMD-18T vector (Takara) and transformed into the strain TG1 of *E. coli *. Positive clones were subsequently chosen for sequencing.

### Oligonucleotides

Oligonucleotides used for construction of the cDNA library, northern blot analyses of novel snoRNAs and the primers for reverse transcription and RT-PCR experiments are not shown (see Additional file 7).

### Database accession numbers

The sequences of all snoRNAs determined in this work have been deposited in the GenBank Nucleotide Sequence Databases under accession numbers EU780925 - EU780999 and EU526091-EU526095.

## Abbreviations

snoRNA: small nucleolar RNAs; rRNA: ribosomal RNA; snRNA: spliceosomal nuclear RNA; tRNA: transfer RNA; UHG: U snoRNA host gene; SL RNA: spliced-leader RNA; pre-rRNA: precursor ribosomal RNA; bp: basepairs; dNTP: deoxyribonucleoside triphosphate; scaRNAs: small Cajal body-specific RNA; RIP: Repeat-Induced Point Mutation; gas5: growth arrest-specific 5; UTR: untranslated region; RT-PCR: reversed transcript PCR; cDNA: complementary DNA; CDS: coding sequence.

## Authors' contributions

NL, LHQ and HZ conceived the study and contributed to manuscript writing. NL performed the experiments. YTL assisted in partial experiments of Northern blot analysis and mapping of ribose methylation. ZDX and DGG assisted in the computational searching for box C/D and box H/ACA snoRNA genes, respectively. CLC and JHY assisted in the data analysis of box C/D and box H/ACA snoRNA genes, respectively. CHY and PS helped to draft the manuscript. All authors have read and approved the final manuscript.

## Supplementary Material

Additional file 1 **The sequences and accession numbers of the box C/D snoRNAs identified from the *N. crassa *genome**. The data showedall the box C/D snoRNA sequences identified from *N. crassa*. Click here for file

Additional file 2 **The sequences and accession numbers of the box H/ACA snoRNAs identified from the *N. crassa *genome**. The data showed all box H/ACA snoRNA sequences identified from *N. crassa*. Click here for file

Additional file 3 **Secondary structures of partial *N. crassa *box H/ACA snoRNAs**. The figures present the secondary structures of six representative box H/ACA snoRNAs from *N. crassa*. Click here for file

Additional file 4 **Potential base-pairing between box C/D snoRNAs and rRNA (A), snRNA (B) or tRNA (C)**. The data showed the functional analysis of the *N. crassa *box C/D snoRNAs. Click here for file

Additional file 5 **Potential base-pairing between box H/ACA snoRNAs and rRNAs**. The data showed the functional analysis of the *N. crassa *box H/ACA snoRNAs. Click here for file

Additional file 6 **Strategy for construction of the specialized cDNA libraries enriched in *N. crassa *box C/D and box H/ACA snoRNAs**. The figure shows that the strategy and pipeline for construction of the *N. crassa *box C/D snoRNA library (A) and box H/ACA snoRNA library (B). Click here for file

Additional file 7 **Sequences of oligonucleotides and primers used in this study**. The data listed all the sequences of oligonucleotides and primers used in this study. Click here for file
