# Supplementary material for: SnoRNAs from the filamentous fungus Neurospora crassa: structural, functional and evolutionary insights
Source: BMC Genomics. 2009 Nov 8;10:515. doi: 10.1186/1471-2164-10-515 (PMC2780460; doi:10.1186/1471-2164-10-515)
Supplement: Additional file 1 — The sequences and accession numbers of the box C/D snoRNAs identified from the N. crassa genome. The data showedall the box C/D snoRNA sequences identified from N. crassa. [file 1471-2164-10-515-S1.pdf]

Additional file 1. The sequences of the box C/D snoRNAs identified from the *N. crassa* genome.

All the box C/D snoRNA sequences determined from cDNA library and computational search are in uppercase letters. Lowercase letters indicate these nucleotides were not cloned owing to the library construction strategy. Conserved box elements are boxed, and predicted functional regions are shaded. In Nc CD11, predicted functional region upstream of box D' can guide two different methylations with single antisense element and are shaded and underlined, respectively. In cases where the termini of the molecules were not cloned, we provide sequences spanning a region 5 nt upstream of the C box and 2 nt downstream of the D box.

snoRNA	sequence	accession number
Nc CD1	uaauaUGAUGACUUGAGUGCAUUUGGCUAGAGUAUCGCGCCUGUCACGGCUGGUCAACCGUUCCGGUCCCAGUUUGUGUAGGGAGCUUUCCAUGAUGAAAAAUCUCAGCUACCUUACAAUUUUCGUCUGAaa	EU780925
Nc CD2	ucaGGUGAUGAGAAACACAAUAAUAGAGUCAAGCUCUGAAGUGGUGCCGGGGUAAAUCUUCGCGCAUCACCCUUGAUGACUCUUAUUGAAUACGAUCUGAUG	EU780926
Nc CD3	aguGAUGAUGACAAAACAUAAAAUUAUGAUAGGAAGAGGAUGAAUUCUCCCACAGAGGAGAGCAUGAUCUCACCAUACGUUAAUCUGAAGC	EU780927
Nc CD4	uuuuAUGAUGAGUCGCUUUACACAUGCAUGGCUUUCUGAAGUCCCGUGACAAGGACUCCUUGUUGAUAGACAGCAACUUCUCUGAuc	EU780928
Nc CD5	ugucCUGAUGACACAACCAUAAACCGCAAACUAGAGUCUUUGAUCCUACGGGAAAUGCAACUAGAUAUGUACGUCAGUCUGAAU	EU780929
Nc CD6	uauGUGAUGAGACGAUAUUCACACUGUUUGACCCUCCUUCGGGUUGGAAUGAACACUAAACAAAAUCUGAUUUAAUGAGgua	EU780930
Nc CD7	uuccaUGAUGAUCCCUUUAGAUGACGAGUCUUAUCGGGGAUUCCCCAUGCAGACAACCUUUUUUUUUAAUACCAUUCAUGCAUUUCUGAaa	EU780931
Nc CD8	uauccUGAUGAUACACACUUUAUACUUUAUUCUGGUACCUCUUAUGUUAAAGUUCCUUUUUGGGACUUGAUGAAUUAAACGGACUUCUGAaa	EU780932
Nc CD9	cuccuUGAUGAUCAACGUACAAAAUAGGGAAUUCCUCAAGCUUAAUACAUCAUGUGGAAUACUUUACUAGUUUCAGCCUUGUCUGAuu	EU780933
Nc CD10	tcctGUGAUGAGAGUAAUUUGCGAUUGAUGCUGCUAUGGUGGGACCCUUUCGCCGGGUGUCCUAUACCUGGUAGUAUUUGAUAGUAUAUUCUUACAUAUUCUGAGUUU	EU780934
Nc CD11	caaucUGAUGAAUCAACUUACAUUCCAUGCUAGUCCUGAAUCUUUAUGUAGAUACAUGUACAGCCUUUUCAUCUUUCGACUGAUu	EU780935
Nc CD12	gcuucuaUGAUGAAACGGCACUGGGCUCCGAUUUUUCCACAUGAGCACGAUAUGCCAUUUUUGCGACAGGCCUAGAAAACCUCGUCCACUGAuc	EU780936
Nc CD13	guacAUGAUGAUAAAAGGGAUUCCUAUGCCUUCGGGUGAUGAGGUAUUGAUACAGCCAUAAGUGGACUGUACCAAUUGUUCCUUCUGAga	EU780937
Nc CD14	caacaUGAUGAUCGCAUUUAAUGGGAAUCUCUCUGAUAUCCAUGAGGAGAAACUCUGCAUCUUAUCUCGUAAAAGCCUGAac	EU780938
Nc CD15	uuuUGUGAUGAUUCUUAGCUCUAGAUCUGCCAGCUGCCUAUCAACAGCACGUGGAGACAACUUCUAAUGUCUGAuc	EU780939
Nc CD16	ucGGAUGAUGACAACUUCUGAAAUGUCUUCUUUUCCCAACUGAAUCGACCAUGAACGAAGACUAGUCUGUUUCGGGCUUCCUCCUACGGGAGUCGGCCUGUU	EU780940

	GCUUGCCAGUAAUUUUUUCUGAcu	
Nc CD17	uccugUGAUGACCUUACACGAACAAUCCAAUUCUUACCUAGCCCAUGGGGAC UCCAAUUUACUCUAGAAUUACCCUGAga	EU780941
Nc CD18	ugcgGUGAUGACUCCUUUUGUGCUUAGAGCAAUAUAUCCUUCAGUGGAUCGU UCUGUGUUAGCCUCUGUAUUAGACUACAACGGUAUCUGAGc	EU780942
Nc CD19	ucauaUGAUGAAAGCAUACGCAAAAUUCACAGACCUGUUCUGAAAAAAAUAG UGUUGAGAAACUGCUUAGCUCACUGAUU	EU780943
Nc CD20	acgGAUGAUGACUUAUUCUUCUUCAAACAACUGAAUCUCUAUGUUGAACAC UUAAUAUAUCAAGCUCAACAGUCUGACa	EU780944
Nc CD21	uuccaUGAUGAUCCGACAACACAAAGACAAGCAUAUGUCAGAGCUUUUGCGA CGAUGACACCUGUCAUGUCUGAuc	EU780945
Nc CD22	uaCUAUGAUGAUCCACAUCUUGAAUACUUAUCUACAUUGUCCAAGCUUCGG CGUUGACGAUAAAUACCUCUGACAUUCUGAuc	EU780946
Nc CD23	cgacCUGAUGACCGAGACAUUUUUCAAACAGUUAUCCCUAUCUGAAAUACGG UGUUGACUUGGUUCUAACUUCUGAcu	EU780947
Nc CD24	uuuccUGAUGAUCAACUUUGCUUUUGCAAGUUGGAAUUACCGAUUGAUACCC AAUGAAAACAUAAAAUGGCUGUUUCUGAuu	EU780948
Nc CD25	cuucAUGAUGAUCUGCCAAAACAUUAGAACCGUUGCGACUGACACGCGCUUG CGCGUGCAUGAUCACACUAUGCUCCAUUUCUGAac	EU780949
Nc CD26	uucuAUGAGGAGAUUAUUCGUGUCGCCCCUAUCAUACACAAUUAUGGUGAU AGACACCGACAAGCCAAACGUCACUACUGAuc	EU780950
Nc CD27	ucgaaUGAUGAGCAACUUUGCGUUAAACCCAGCUCACUCAGAGGCAAAAUAA UUGUCGAUGCUGAGAACGCGCUACAUCUACUCCUGACA	EU780951
Nc CD28	caauaUGAUGAUUUCUCGAAAGAGAGGCUGUUUAUGAUCAACAAUGAUUACC AAAAUACACCUAUACAUUGUUCUAUUUCUGAuc	EU780952
Nc CD29	cugcGUGAUGAAAAAUAUUUUGCUGCGUUCUUUAUGACAGACCAGCCUUCUC UUUUUCAAGAAGGGUGGCAUGGCCUGCUUGAUUAUCAACUACACUUGAUC UGAua	EU780953
Nc CD30	cuuuaUGAUGAUCUUUUUAUUGGAGCUGUCUGUAGCCUGCUAUGUCUGGCGU UUGAUGACAAACUUUUACAUACGCUUUUUUUAUCUGAug	EU780954
Nc CD31	uacaaugaugaauaaaauuuaucacgacggucuacauaggauaucgcuucggcuUUAUCCCUUUGAUG AAAAACGCCGAUGACACGGCACCCGAACUGAgu	EU780955
Nc CD32	gacctGGAUGAUGUGCAAUUUCAGCAUGUAUUAUCUGAUCGUGGAACAAUAU CCACGAAUGAUGUCCAAAUUAUUAGCUCUAUCUGAcg	EU780956
Nc CD33	uuccaUGAUGAAAAUCUAUCCCAUUUUUUGAUGACGCAGGUCGGCGCCCCGUC AUGGGGUUGCUGUCUGCACCAUAAUGAUUACCUUUAUGUUCAUAGUUACUC UGAcu	EU780957
Nc CD34	ucucaUGAUGAUCCCAUAACCACAGUUCUCGCGAUAAUCUCUGAGCGAUCGAA CAAACAUGCACUACCAUCUGAcu	EU780958
Nc CD36	cuuuaUGAUGAACCCCUGAUAGGGACAUCUUAUGCCAAACGCAAAUGAUGUG AAUUUGGGCUCGGUAUUUUAGUAGAUGCUUCGAGUUUCUGAUC	EU780959
Nc CD37	ccuuaUGAUGAAGUUUUCCUACAUUCGCAGUUUCCACUCGAGACCCCACCUAC GAGCCUUUCUCGGGGUCAGAUGAGGGCGAAGUCUUCCUUGGAUGUCUGAuu	EU780960
Nc CD38	gucCAUGAUGAAACAAAAUAUUUUCAGUUCUGCUUCUGAAUAAUAAUGAGC	EU780961

	GAUAACCAAUUAUACCAAAACCUUCUGAUAUG	
Nc CD39	ucauaUGAUGAACUACAAGAAAAUUCGAUUAGUCUUGAAGACAUACCUCAUG<br>GUUACAAAGCCUUUUUUCAACUGAGC	EU780962
Nc CD40	uuuuAUGAUGAGUUUGUUCCAAUUCAUGAGUUUUUCUAAGUUGGGCCCAGC<br>GUUUUGCUGGGCUUUGAAGUGAUUAUCGUUAUACAACAUAAGCCAAUCCUC<br>UGAaa	EU780963
Nc CD41	uuucaUGAUGAGAAAAACAAUGUUCACAGACCUGUACCGAAACACUAUGUGG<br>AAAAACUUGUCCAACCGCUAUCUGAga	EU780964
Nc CD42	CACCCUGAUGAAAUAUUCUCGGAUGUAAAAUUUUACUUUUGUCUGAAAAG<br>CGCAAAAACAAUGUUGAGAUUUCCGUUGCCUUCCUUCUGAUC	EU780965
Nc CD43	UUCUAUGAUGAUUGUUCAGCUGGCACCAGUCAUAGGGUGACUUGUACUCCC<br>UCCCUUGAUGACAAACGCUGCGGUCUACCAGGACCAUCUCUGAUC	EU780966
Nc CD44	UUUCAUGAUGAAACAACAAACGUAUCAAUCUGUCAAUCCCCUGAUUCAUAG<br>UGAUGAUAAAUACGGCUGUCCAAGGCUUCUGAUC	EU780967
Nc CD45	AGCAAUGAUGUCGUGGGUAGGUUUGUAACGAGUGGCGGGCGGCCUGACAG<br>UUGGUGCCGAUGUUGUGUUGGGUUGGGGGUCGCGGUCGGGCAGCGUCUGA<br>CC	EU780968
Nc CD46A	UCGAAUGAUGAACACACUGGUCCGUGUUGCUGAGCAGCUUAAGACCCUGCA<br>AAUGAUUAACACAACAGCACUUUUGUGCACUUCUGACU	EU780969
Nc CD46B	UCCCGUGAUGAUCGAAUUGGUCCGUGUUACCAAGCAGACUUAAGACCUCUG<br>CAGAUGACGACGAAACCAGCAUUUAUGCGACUCUGACC	EU780970
Nc CD47	CAACAUGAUGCACAACAUACCAUAGCGACUAGGUUCGCCGAUGCUGUUGCU<br>AAAUACCAUCUUUCGGGUCUGACA	EU780971
Nc CD48	UUCCGUGAUGAUUUACAAUCAUAAGCAUAGUUCAUCUGAAUUAUUCAUGA<br>AGACAACUACGUAUUUUAUAGUCUUUCGCUCCCAUCUGAUU	EU780972
Nc CD49	CCUCCUGAUGAUAAACCUAUAAAUCAUAACUUUCGUUCCACCAUAUCGACC<br>UAUGUGUCAUGAGGAAACAAAUACACUGAAAAUCCUGAUU	EU780973
Nc CD50	AAACAGGAUGACCAACGGUCACCACCAAGAUCUGUGGGUGUAUGUAUGUA<br>UGUAUGUAUGUAUGUAUGUAUGUACCGCAGUGGGUGGGUAGACUGACC	EU780974
Nc CD51	CACCAUGAUGCAUGCAUAUUUUUCUCAAAAUAGUAUUUGCUACUUCAGAG<br>CCCUCGGGCCAUGAGACCAACAUCACACGCACUGAUU	EU780975
Nc CD52	AAUCAACUAAACUGGGUAUGAUGAGCCCGCUAAGAACACCAGCGCUUUUGC<br>UCCUCCAAGGUCUUUUUUUUCGAAAGAAAGAAAGAUCUUCUUAGGGGGAC<br>GAAUUCAUUUCGCUGAUAUGCCCUGGCACAAAACUCACCACUCUGAAAGGG<br>GACAAUUUGAGGAAAUCUCUGACCC	EU780976
Nc CD53	ACAAUGAUGACAACAGUCUAUCCAUUCAACGAUAUGUACGGGCCGAGCCAU<br>CCAUCCGCACUGGAGCGUCUUUCGGGGCGUGAGGACUAGUUGUGGUGUCUC<br>CCUUCGGGGUGUCAUUUGGCUUCCGUUGUCCGAGUCUCCUGCGCACCGUUU<br>UGGGGCGUGGGAGGCUUGGGCCGGCUUUGUAGGGCUGAGUUAAAACGGCUU<br>GUCUGAUC	EU780977
Nc CD54	aUUCCAUGAUGACGCACGCGGGGCCAUCAGAUGUCUUUUUUCGCCUCUGGG<br>UCCAACUCCCAUGGGUGAAAAGGCAAACGCUGACAAACACCGCGUUCCAAC<br>AAUCUUCUCAAACACUCugaaau	EU780978
Nc CD55	UGAGAUGAUUAGAACACGAGUUUCAAUCAUGCACCACGCAUAAGCCUUCCG	EU780979

GUGAACCUUUGGGUUGGCUUUCGGGUCGACCUUUGGGCUAGCCUUCGGGCU

CUUUGAUGAGAACCAACCACUUCUUUAUGAUUCCU
