# Supplementary material for: SnoRNAs from the filamentous fungus Neurospora crassa: structural, functional and evolutionary insights
Source: BMC Genomics. 2009 Nov 8;10:515. doi: 10.1186/1471-2164-10-515 (PMC2780460; doi:10.1186/1471-2164-10-515)
Supplement: Additional file 2 — The sequences and accession numbers of the box H/ACA snoRNAs identified from the N. crassa genome. The data showed all box H/ACA snoRNA sequences identified from N. crassa. [file 1471-2164-10-515-S2.pdf]

Additional file 2. The sequences and accession numbers of the box H/ACA snoRNAs identified from the *N. crassa* genome.

All box H/ACA snoRNA sequences determined from cDNA library and computational search are in uppercase letters. Lowercase letters indicate these nucleotides were not cloned owing to the library construction strategy. Predicted H and ACA-like motifs are boxed.

snoRNA	sequence	accession number
Nc ACA1	UCGGCGUAUAACUAGGCCGAGGUCUCACUACGCUCCACGAUCUUAGUCUUUUAAGUCGCCGAUAUAUCAAAUGCGAGAGUCUUACACGUUACUGGAGUCACCAAUGAUAUCUAAUGUUUAAACUGUCGCGACAUUU	EU780980
Nc ACA2	GGCACCAUUCCUUAUCAUCACGACGUUUCUCUUCCUCCAAAACUGUCCUGAUCAUUUAGUCCGUGCCGAGAGGAACCACCAUGAGCAGACGAAUUCGCUAGCGGUACCUGCCUAUAAACGCUCUACCGGGUUGCUGUUAGAUAGAAUCUGCUUACAUAU	EU780981
Nc ACA3	AACGCGUCCUCAGACAGUGCCGAACAUCGCAAAUGCACAUCGGCUCCGGCAUUCUGUCGGGGUUCGAACCGUGUCCUAUGGUGAUUCUGCGAUUUCGCGACGCGGAGAAGAAGGGGAUUGAUUUCUUCAAAUGGCUCCAUAAGAGCUGCCCAUGUAUGUUGUCUGCCUUUGGGAAGAUGACCAAAAUUACUAGGGCGGCGUCAGAGGUUCCACACAu	EU780982
Nc ACA4	UUAUUGUACUACGGCCACUUAUUGUAGGGGGAUAUUGGUUCGCAAACUGUCCACGCUUUCGGGUAUGGCUUUUGCACGCUCUACGAGUGUUCCCCUCUAUCAUGUGUCCUAGAUAGAUUUUCUUUGGCUCCGUUUAUGGAUACACGCUAGAUGUCCCUUUCGAGGGCGUCUCUGAGCAGUGAUUCUUACCGCCGGUUGCCACAucu	EU780983
Nc ACA5	AACCACUCAACGAAGAGACGCGGUUGCUGCUGCUGCUCUGCAGCCCAACAGUAUCGCGGGAGUCUUGGGGGGCCUUUCAUUAGGUUUCUCCGAAGUUGAGUGGCAGAUUGAUGCCACAAACAGCCCUUCUGUAUCUCCUGCCUUAGAAUCACCACAUGUGAGGGCGGGUCGAACGAUCGGCACAUUU	EU780984
Nc ACA6	ACAACACAUAAAACAUAUGUUCCUUCAUAUUCACUCCGGUUUCCGUCUACUGCAAUGACAUUCUGGAGGGGAAAGUGGAGGAUAGAAGAGAAUUUUUCAAGCUCUUACCCUCUACCUUUGUUCUUCUCUGUCGAGGAGUAGAGAAAUAUUUUGCCUGGGACAUUU	EU780985
Nc ACA7	GAUAGUCCGGGUGUUUCCAGCCAUAGCAUUGGGUUUCUUCAGUGAGCAGUGCAUACGUGGCAAGGGGGCUUUGGUUCCCAUGGACGCUCGAAGAAGAAAUGAUCCUUCGUUAAUCUCUAACGUCAGUUAAUCAGUCUGGCGGUGGUUUCAUUCUGAGAUCUACAUUU	EU780986
Nc ACA8	AGAAAGUUGCACCCGGGGACGCAGAAUAUAGGCCCAAUGACGAAUUCUACUGUUUCAGACAGUCGUUGGGUUGUAUUAACCGGGGAGAUUAAAACUGAGAGUCUCAAGAAUUGCUAUGAAUGCGUUGCCCUUGUUUUCUUCGGAAGUCAAGGCUCCGCGAUGAAUACGCGAUCCCGUUUGGCUUCGACAUuu	EU780987
Nc ACA9	AUCCUCGGAAUAUCUGCCCGCAUAGUGCAAUGCAUACAACUAUUGCGGUUCCUGGAACCGAGGAAGAUUACCGCUACAUCUCAGCCGCCCAUCAUUCGCAAAAUACCGGGUGGGCUGCGGUGUGGCCAUGGUGGGAAUGCUUUGGAUGACCCGAGCAAUAUACUCUGUGUGCUCGGUGUGGUUUUUUAGCGCCCUACAUCU	EU780988
Nc ACA10	aaguugucacuggcuccugcuuCCAUGUCGUGGCAACUAGUCUGUGUGCAAAGAUCGAGCUUCAUUGUCGCCCUGAGAAGAAGGCGUCUUGUCUCCUUCUCGUUUGGGACUGGCAUGGAGCGCCAAUCCGAGCCUUUUUGGACUGGGUUCUUUGGAGCCUUUUCCAUCUUUC	EU780989

	GCUACUCCUCACGCCUUCGGGUGGAUAUCCACUUGCUUUCGGGCAGACAUGUCUU<br>UGGGCGCAUGCUUUCGAGUGUGCACUAUGCCGUCGGACGGAUGGGAGAAGGGAG<br>UUUGCUGUCUUUCGAGAUUGGAACAUGC	
Nc ACA11	ACAAUGUCCACGGCAUUCCUAUUAGAGUCUCGGAACAAGACCAUCAUUUCUUGUU<br>CAGGCCCAUGAGGUCUCUCGCGUGCUCAAGGAUCGCCCAGAGGAAUACCGCACUU<br>UCCGUUUAAGUUACCUAACACCUUCACGGGCGUUUAGGUGGACAUAUCUGGACAA<br>GUGAUACAuuu	EU780990
Nc ACA12	ACGGACCUGUGCCCAGCGCUCCUCUGCCGCCAGCUCGUGCUUGCACUUGUAGGCA<br>GCUAGGGUUGAGUGCUGAGGCAGUUCGGCAACCAAGGAGCCAUCCAGGACUUUAC<br>GUUCUUCCAUGAUGUCAAUUUCCAUCCGACAGGUCACCAGAAGAACCGAACUCCG<br>GUGUGAACCUCGUUCGCCGCAAGGCUUGAGCUUGGGGAGCGCCGGAUGAUGUAG<br>GGGAAGUCAUAUUCCAAUCCGUUCGCCGAGGACGUCGUCCUAUGAGGCUGGGGGA<br>AACCAUGACAAUUGCCACAuuu	EU780991
Nc ACA13	AAUUUUUACAACGCAUUAUAUCACAUUCUCGGAAUGCAACUUGGAUGCGAGGUC<br>CCCGGAUUCGUUCGGAGCUGACCCAGGACCAAUUCAUCGAUUUCGAACAGAAUGG<br>AGAACAGAUACCAGCGAACAAUCUUGGGUGCGGUGCUUUCCUUGCCCUUCGUGGC<br>UCCAUGGAAAGGGUCGCAAUGAUUGGGCGUACAUACCUACAucg	EU780992
Nc ACA14	acauauuuucccuucgccugcaggcagguaacguuuuucucagcuucggcuggggagauguucaccugucggauuugucuc<br>uuuuucuuucucuagaaaaagaacucaaUGACUCUGUUCGCAAGACAGGAAGAAGUAGAACAGA<br>UUUCCACAGCGCGCUUUAUAAUCCUGAUCGGCCUUCUUUCUUUUUUUCUCCUAAU<br>GGGUCUUGUUCAGGUUCGCUUGGUGCGUACAUCA	EU780993
Nc ACA15	gcaaggaugcucucucuacaccgccauguguuggagaguggccuuguuacuaacuacauuaaauacugguggcuccgcauagc<br>ggcuugcugaggguuacucuuguaccauacucccGAUGGGUCUUCCUCCGUUCCCGGCCUCCUAAA<br>GUAAUGCUUCUCCAUCUCUUCCCUUUACACUAGUCGCGGACGUCGAACAUUCUAG<br>GUCCGUGAUCGUGGGGUGGUUUCGACAUUU	EU780994
Nc ACA16	CUGGAGCCUCAAGAAGGAGGGUGGUGUCCUUGGUGAUGUUGCUCCCACGAUCCUU<br>GCGGCUAUGGGUCUCCCCCAGCCCGCGGAGAUGACUGGCCAGAACCUUCUUGUCA<br>AGGCCUAAAGUAGAACUUGUAAUGAUUGGAUGGCGUGAUGAAAUAAGUAACAUA<br>GAUUAUAGACGGGAAUGACAACG	EU780995
Nc ACA17	UAGCACGGAUGUCAAAUUGCAAUCUAGUCGCGUUCCCUCUUCAUGAGAGAACCAU<br>CAAUGGCUAUGGUUGUGCCGAAGAUGUGCUGAAAUCAAACCUCACUCUGCAAGU<br>AGCCUGGUAUCCGGGCACGUCGUCAUCAGGGACUCACUUUCUACGGCAAGGAGUC<br>UGUUGCCGGCGGAUUGCGUACAUUU	EU780996
Nc ACA18	AGCUUCAUCAUUAUGCUAGAGCUUGUCGCCUUCAAAAUAACCCCAGGGCGCGCAA<br>GUGUGGACAAUGAAGCAAGAAUAAAUGUACGGCUGGUUCAUUCCUUCAAAUGGC<br>CACCCUCCGUCAAGUCAUGAAUCAUCUUGUCUGCCCGUCCCGGGUCUUGAUGGUA<br>UGUGCUUGCGGAUACAUAUCCU	EU780997
Nc ACA19	AUCCAGCGAUGCUUUCACUCGAGAAAGCCGGAGUCGAUUUCCAUCCCAGGAACGG<br>UUCUGAACGGAGCGUUGGUAGAAAGAAUUUCCAUUAUAGAAGGCUCGUUGUUCC<br>GACUCGUUUUACGAGUUGACUUCCGCAAGGUUGGUGGGCAAUGAUGCAAGAGAA<br>CCCUUGGGAACAUUU	EU780998
Nc ACA20	cauuuuuguacggcaaccaacuaaauaaucgcuauuuuGUACGGUUAUUGUAUAGAAAUAGCCGUAU<br>GAGGCGGUUUAUACGGCAACUGUACGAGAACAAACGCAGCCUUAAAUGGUGCAA<br>GCGGGAAAGCGCUGCGGUGUGAGUGAGGAUGUGACACGC	EU780999
