# Supplementary material for: SnoRNAs from the filamentous fungus Neurospora crassa: structural, functional and evolutionary insights
Source: BMC Genomics. 2009 Nov 8;10:515. doi: 10.1186/1471-2164-10-515 (PMC2780460; doi:10.1186/1471-2164-10-515)

Additional file 3. Secondary structures of partial *N. crassa* box H/ACA snoRNAs. Box H and ACA motifs are boxed.

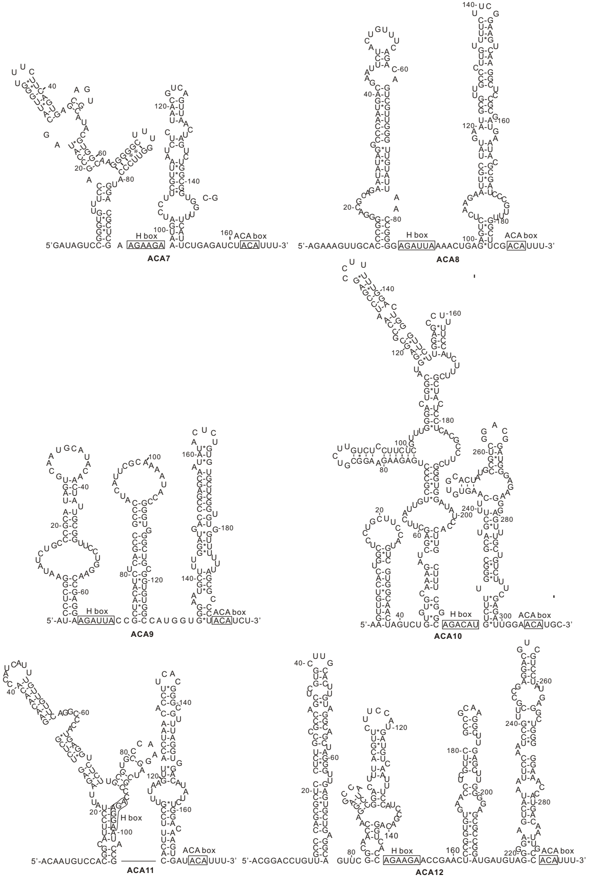

Supplement: Additional file 3 — Secondary structures of partial N. crassa box H/ACA snoRNAs. The figures present the secondary structures of six representative box H/ACA snoRNAs from N. crassa. [file 1471-2164-10-515-S3.pdf]
