# Supplementary material for: SnoRNAs from the filamentous fungus Neurospora crassa: structural, functional and evolutionary insights
Source: BMC Genomics. 2009 Nov 8;10:515. doi: 10.1186/1471-2164-10-515 (PMC2780460; doi:10.1186/1471-2164-10-515)
Supplement: Additional file 4 — Potential base-pairing between box C/D snoRNAs and rRNA (A), snRNA (B) or tRNA (C). The data showed the functional analysis of the N. crassa box C/D snoRNAs. [file 1471-2164-10-515-S4.pdf]

Additional file 4. Potential base-pairing between box C/D snoRNAs and rRNA (A), snRNA (B) or tRNA (C). Box C/D snoRNA (top) and target (bottom) are shown. Predicted D or D' boxes are boxed. Star indicates a G/U match. Black dot represents the fifth position before the box D or D'.

A.

```
Nc CD1  D'
5'-GCAUUUGGC[UAGA]-3'
3'-CGUAAACCGA-5'
26S-Am2242

Nc CD2  D
5'-UAGAGUCAAGCU[CUGA]-3'
3'-AUCUCAGUUCGA-5'
26S-Um2379

Nc CD3  D'
5'-UAUGAUAGGAAGAGG[AUGA]-3'
3'-AUACUAUCCUUCUC-5'
26S-Um2840

Nc CD4  D'
5'-UUACACAUGCAUGGCUUU[CUGA]-3'
3'-AAUCUGUACGUACCGAA-5'
18S-Cm49

Nc CD5  D'
5'-CAAACUAGAGUCU[UUGA]-3'
3'-GUUUGAUCUCAG-5'
26S-Um2383

Nc CD5  D
5'-UGUACGUCAGU[CUGA]-3'
3'-ACGUGCAGUC-5'
26S-Am856

Nc CD6  D'
5'-UAUUCACACUGU[UUGA]-3'
3'-AUAAGUGUGAC-5'
26S-Um2687

Nc CD7  D'
5'-UUAGAUGACGAGU[CUUA]-3'
3'-AAUCUACUGCUC-5'
26S-Gm2250

Nc CD8  D'
5'-UUCUGGUACCUCUU[AUGU]-3'
3'-AAGACCGUGGAGA-5'
26S-Gm2751

Nc CD9  D
5'-AGUUUCAGCCUUGU[CUGA]-3'
3'-UCAAAGUCGGAAC-5'
18S-Gm1122

Nc CD10  D'
5'-GGGACCCUUUCGC[CGGG]-3'
3'-UCCUGGGAAAGCG-5'
26S-Am3264

Nc CD10  D
5'-UUCUUACAUAUU[CUGA]-3'
3'-AAGAAUGUAUAA-5'
26S-Um1039

Nc CD11  D'
5'-AUUCCAUGCUAGUC[CUGA]-3'
3'-UAAGGUACGAUCA-5'
18S-Am793

Nc CD11  D
5'-CUUUUCAUCUUUCGA[CUGA]-3'
3'-GAAAAGUAGAAAGCU-5'
26S-Am356

Nc CD12  D'
5'-GGCACUGGGCU[CCGA]-3'
3'-UCGUGACCCG-5'
26S-Cm2159

Nc CD13  D
5'-CCAAUUGUUCCUU[CUGA]-3'
3'-GGUUAACAAGGAG-5'
18S-Am538

Nc CD14  D'
5'-UGGGAAUCUCU[CUGA]-3'
3'-ACCCUUAGAG-5'
26S-Am2288

Nc CD15  D'
5'-UUAGCUCUAGAU[CUGC]-3'
3'-AAUCGAGAUCU-5'
18S-Am159

Nc CD16  D'
5'-GUCUUCUUUUCCCAA[CUGA]-3'
3'-CAGAAGAAAGGGG-5'
26S-Gm2357

Nc CD16  D'
5'-UUCUUUUCCCAA[CUGA]-3'
3'-AGGAAAGGGG-5'
26S-Gm1907

Nc CD17  D'
5'-GAACAAUCCAAUU[CUUA]-3'
3'-CUUGUUAGGUUG-5'
26S-Gm2875

Nc CD17  D
5-'CUCUAGAAUUACC[CUGA]-3'
3'-GAGAUCUUAAUGG-5'
18S-Am154

Nc CD18  D
5'-ACGGUAU[CUGA]-3'
3'-UGCCAUAGACU-5'
18S-Cm1004

Nc CD19  D'
5'-UCACAGACCUGUU[CUGA]-3'
3'-AGUGUCUGGACAA-5'
18S-Gm1423

Nc CD20  D
5'-UCAAGCUCAACAGU[CUGA]-3'
3'-AGUUCGAGUUGUC-5'
26S-Um2372

Nc CD21  D'
5'-AGACAAGCAUAUGU[CAGA]-3'
3'-UCUGUUCGUAUAC-5'
18S-Am28

Nc CD22  D'
5'-CUUAUCUACAUUGU[CCAA]-3'
3'-GAAUAGAUGUAACA-5'
26S-Um1866

Nc CD22  D
5'-UACCUCUGACAUU[CUGA]-3'
3'-GUGGAGACUGU-5'
18S-Um893

Nc CD23  D'
5'-CAGUUAUCCCUAU[CUGA]-3'
3'-GUCAAUAGGGA-5'
26S-Gm2773

Nc CD24  D'
5'-AGUUGGAAUUACCGA[UUGA]-3'
3'-UCGACCUUAAUGGC-5'
18S-Um575

Nc CD25  D'
5'-AGAACCGUUGCGA[CUGA]-3'
3'-UCUUGGCGACGCU-5'
26S-Am846

Nc CD27  D'
5'-UUAAACCCAGCUCACU[CAGA]-3'
3'-GAUUUGGGUCGAGUG-5'
26S-Am2904

Nc CD27  D'
5'-UUAAACCCAGCUCA[CUCA]-3'
3'-GAUUUGGGUCGAGU-5'
26S-Cm2906

Nc CD28  D'
5'-AGAGGCUGUUU[AUGA]-3'
3'-UCUCCGACAAG-5'
26S-Am1845

Nc CD28  D
5'-UACAUUGUUCUAUUU[CUGA]-3'
3'-AUGUAACAAGAUAG-5'
26S-Am1859

Nc CD29  D'
5'-GCUGCGUUCUUU[AUGA]-3'
3'-CGAUGCAAGAAA-5'
5.8S-Am42

Nc CD30  D'
5'-UUAUUGGAGCUGU[CUGU]-3'
3'-GAUAACCUCGAC-5'
18S-Cm584

Nc CD31  D'
5'-UCACGACGGUCUA[CAUA]-3'
3'-AGUGCUGCCAGAU-5'
26S-Cm2917

Nc CD32  D'
5'-AGCAUGUAUUAU[CUGA]-3'
3'-UCGUACAUAAU-5'
18S-Um167

Nc CD32  D
5'-UAUUAGCUCUAU[CUGA]-3'
3'-AUAAUCGAGAU-5'
18S-Am161

Nc CD33  D
5'-UCAUAGUUACU[CUGA]-3'
3'-AGUAUCAAUGA-5'
26S-Am2218

Nc CD34  D'
5'-CAUGCACUACCAU[CUGA]-3'
3'-GUACGUGGUGGUG-5'
18S-Um1265

Nc CD36  D'
5'-GAUAGGGACAU[CUUA]-3'
3'-CUAUCCCUGU-5'
26S-Cm2299

Nc CD37  D
5'-GUCUUCCUUGGAUGU[CUGA]-3'
3'-CGGAAGGAACCUACA-5'
18S-Cm411

Nc CD 38  D'
5'-UCAGUUCUGCUU[CUGA]-3'
3'-GGUCAAGACGAA-5'
26S-Am1114

Nc CD 38  D'
5'-AGUUCUGCUU[CUGA]-3'
3'-UUAAGACGAAG-5'
26S-Am2858

Nc CD39  D'
5'-UUCGAUUAGUCUUG[AAGA]-3'
3'-AAGCUAAUCAGAA-5'
26S-Am897

Nc CD39  D
5'-CCUUUUUUCAA[CUGA]-3'
3'-GGAGAAAAGUU-5'
26S-Am375

Nc CD40  D'
5'-UUCUAAGUUGGG[CCCA]-3'
3'-AAGAUUCAACC-5'
26S-Am2062

Nc CD41  D'
5'-UCACAGACCUGUA[CCGA]-3'
3'-AGUGUCUGGACA-5'
18S-Gm1423

Nc CD41  D
5'-UGUCCAACCGCUAU[CUGA]-3'
3'-ACAGGUUGGCGA-5'
26S-Cm1489

Nc CD42  D
5'-GUUGCCUUCCUU[CUGA]-3'
3'-CGACGGAAGGAA-5'
18S-Am417

Nc CD43  D'
5'-GCUGGCACCAGU[CAUA]-3'
3'-CGACCGUGGUC-5'
18S-Gm559

Nc CD44  D'
5'-UCAAUCUGUCAAUCCC[CUGA]-3'
3'-AGUUAGACAGUUAGG-5'
18S-Um1227

Nc CD44  D
5'-GUCCAAGGCUU[CUGA]-3'
3'-CAGGUUCCGAA-5'
26S-Cm776

Nc CD45  D
5'-CGGUCGGGCAGCGU[CUGA]-3'
3'-GCCAGCCUGUU-5'
26S-U3021

Nc CD46A  D'
5'-UGGUCCGUGUUG[CUGA]-3'
3'-ACCAGGCACAA-5'
26S-Am635

Nc CD46B  D'
5'-UUGGUCCGUGUUA[CCAA]-3'
3'-AACCAGGCACAA-5'
26S-Am635

Nc CD47  D
5'-UACCAUCUUUCGGGU[CUGA]-3'
3'-GUGGUAGAAAGCCCA-5'
26S-Gm785

Nc CD48  D'
5'-UAAGCAUAGUUCAU[CUGA]-3'
3'-GUUCGUAUCAAGUG-5'
26S-Am797

Nc CD48  D
5'-UAGUCUUUCGCUCCCAU[CUGA]-3'
3'-AUCAGAAAGCGGGGGUA-5'
26S-Gm888

Nc CD49  D'
5'-UAACUUUCGUUCCA[CCAU]-3'
3'-AUUGAAAGCAAGG-5'
18S-Am971

Nc CD49  D
5'-ACACUGAAAAUC[CUGA]-3'
3'-UGUGACUUUUAG-5'
26S-Um2682

Nc CD50  D'
5'-ACCACCAAGAUCU[GUGG]-3'
3'-UGGUGGUUCUAGA-5'
26S-Cm1418

Nc CD51  D'
5'-AGUAUUUGCUACUU[CAGA]-3'
3'-UCAUAAACGAUGA-5'
26S-Am1430

Nc CD54  D
5'-CUCAAACACU[CUGA]-3'
3'-GGGUUUGUGA-5'
26S-Um667
```

B.

```
Nc CD11  D'
5'-CCAUGCUAGUC[CUGA]-3'
3'-GGUACGAUUAG-5'
U6-Am47

Nc CD24  D
5'-AAAUGGCUGUUU[CUGA]-3'
3'-UUUGCCGACAAA-5'
U5-Am62

Nc CD26  D'
5'-UUCGUGUCGCCC[CUAU]-3'
3'-AAGCGCAGCGG-5'
U2-Gm183

Nc CD29  D
5'-ACUACACUUGAU[CUGA]-3'
3'-UGAUGUGAACUA-5'
U2-Am31
```

C.

```
Nc CD15  D
5'-AACUUCUAAUGU[CUGA]-3'
3'-UUGAAGGUUAC-5'
Thr tRNA-Um114

Nc CD52  D'
5'-GGUCUUUUUUUU[UCGA]-3'
3'-CCAGGAAAAAG-5'
Leu tRNA-Am43

Nc CD52  D'
5'-AGGUCUUUUUUUU[UCGA]-3'
3'-UCUAGAAAAAAA-5'
Leu tRNA-Am90
```
