# Supplementary material for: SnoRNAs from the filamentous fungus Neurospora crassa: structural, functional and evolutionary insights
Source: BMC Genomics. 2009 Nov 8;10:515. doi: 10.1186/1471-2164-10-515 (PMC2780460; doi:10.1186/1471-2164-10-515)

Additional file 5. Potential base-pairing between box H/ACA snoRNAs and rRNAs.

The predicted box elements are boxed and hairpin domains are schematized by a solid line. Predicted pseudourdylation Sites are denoted by Ψ.

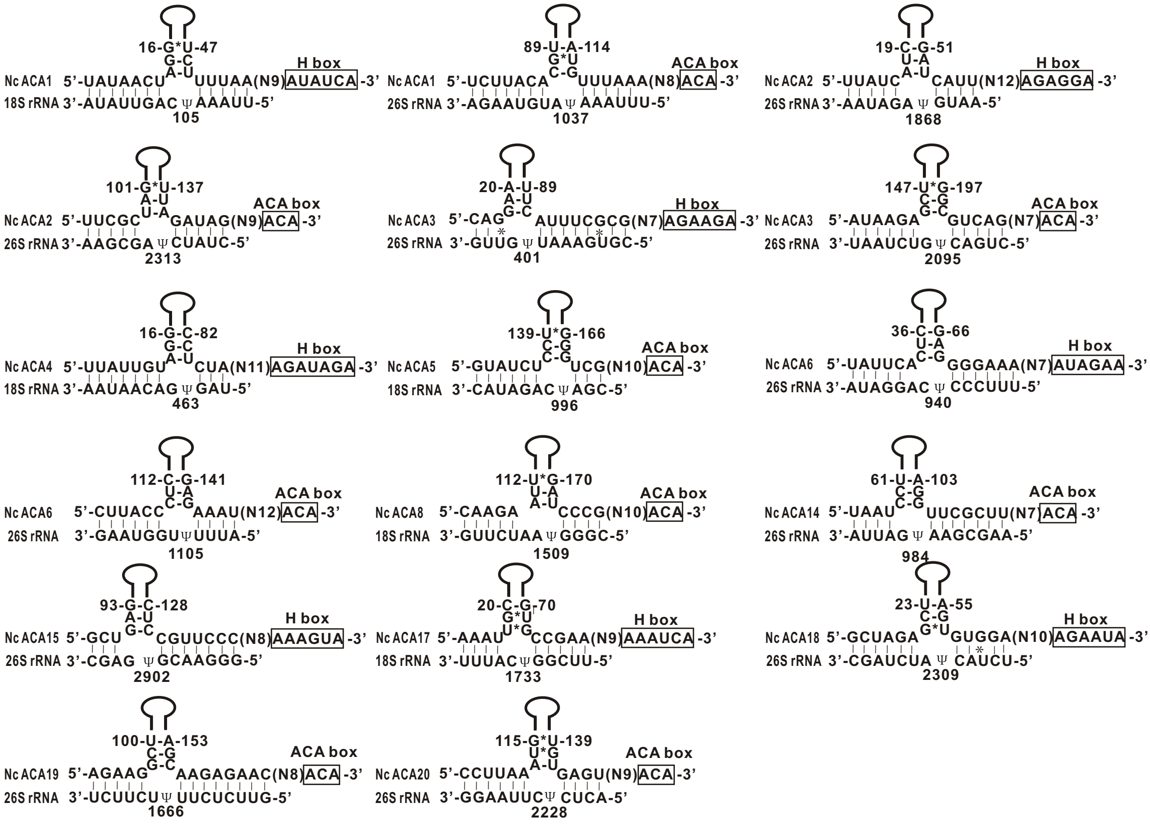

Supplement: Additional file 5 — Potential base-pairing between box H/ACA snoRNAs and rRNAs. The data showed the functional analysis of the N. crassa box H/ACA snoRNAs. [file 1471-2164-10-515-S5.pdf]
