# Supplementary material for: SnoRNAs from the filamentous fungus Neurospora crassa: structural, functional and evolutionary insights
Source: BMC Genomics. 2009 Nov 8;10:515. doi: 10.1186/1471-2164-10-515 (PMC2780460; doi:10.1186/1471-2164-10-515)

Additional file 6.

Strategy for construction of the specialized cDNA libraries enriched in box C/D and box H/ACA snoRNAs

A. Construction of the specialized box C/D snoRNA libraries

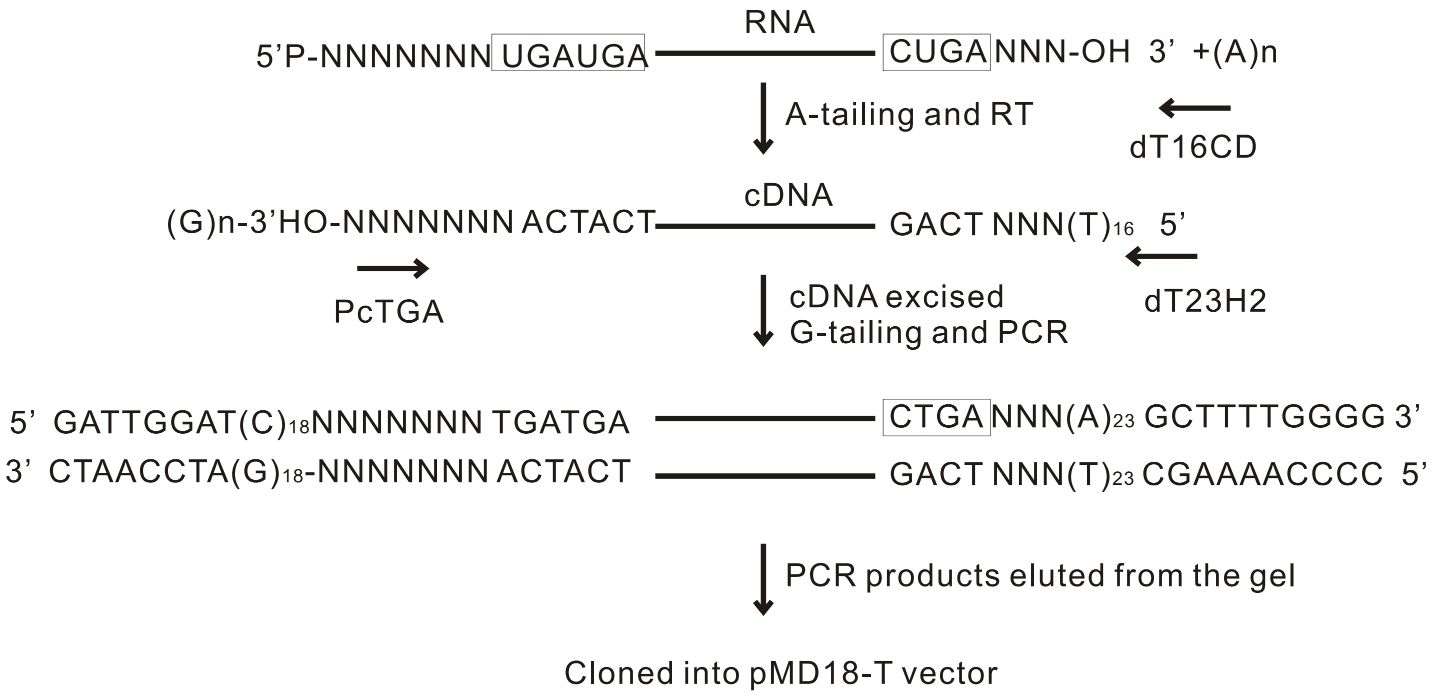

B. Construction of the specialized box H/ACA snoRNA libraries

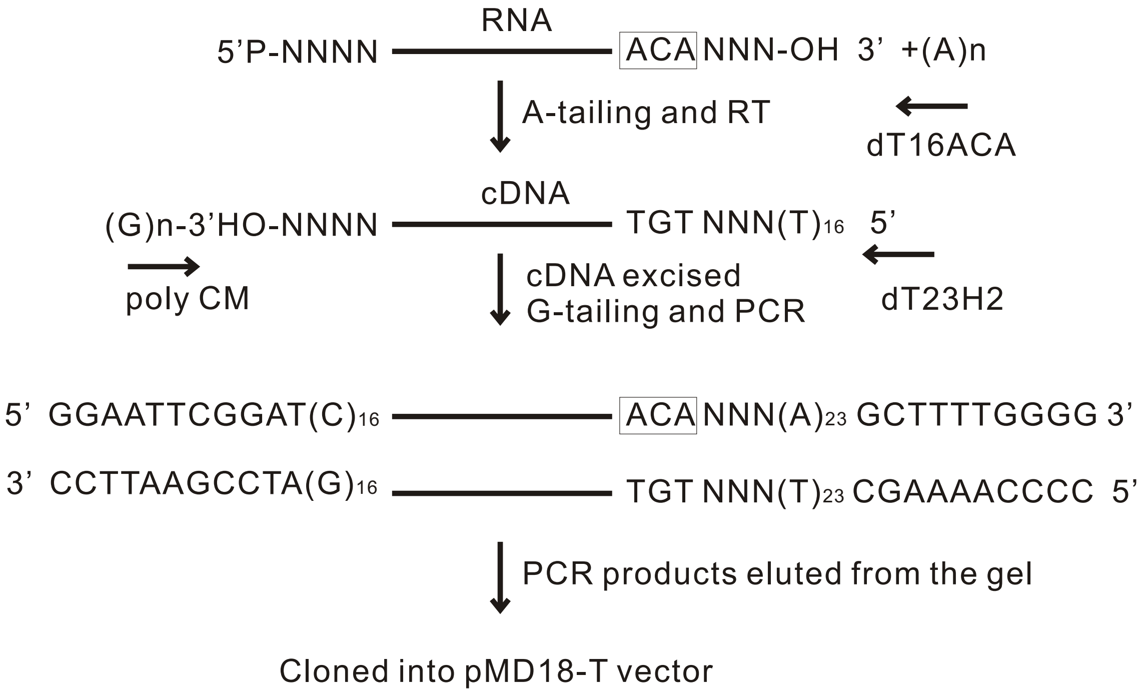

Supplement: Additional file 6 — Strategy for construction of the specialized cDNA libraries enriched in N. crassa box C/D and box H/ACA snoRNAs. The figure shows that the strategy and pipeline for construction of the N. crassa box C/D snoRNA library (A) and box H/ACA snoRNA library (B). [file 1471-2164-10-515-S6.pdf]
