# Supplementary material for: SnoRNAs from the filamentous fungus Neurospora crassa: structural, functional and evolutionary insights
Source: BMC Genomics. 2009 Nov 8;10:515. doi: 10.1186/1471-2164-10-515 (PMC2780460; doi:10.1186/1471-2164-10-515)
Supplement: Additional file 7 — Sequences of oligonucleotides and primers used in this study. The data listed all the sequences of oligonucleotides and primers used in this study. [file 1471-2164-10-515-S7.pdf]

Additional file 7. Sequences of oligonucleotides and primers used in this study.

A) The oligonucleotides used for the construction and screen of cDNA library

Oligonucleotides	Sequence (5'→3')*
dT16-CD	TTTTTTTTTTTTTTTTTNNNTCAG
dT16-ACA	TTTTTTTTTTTTTTTTTNNNTGT
PcTGA	CATTGGATCCCCCCCCCCCCCCCCCNNNNNNNTGATGA
dT23H2	CCCCAAAAGCTTTTTTTTTTTTTTTTTTTTTTT
polyCM	GGAATTCGGATCCCCCCCCCCCCCCCC
P47	CGCCAGGGTTTTCCCAGTCACGAC
P48	AGCGGATAACAATTTCACACAGGA

*N=ATGC

B) The oligonucleotides used for northern blot（N） and reverse transcription (R) analyses of novel snoRNAs were as follows.

Oligonucleotides	Sequence (5'→3')
Nc snR1	TGGGACCGGAACGGTTGA (N)
Nc snR2	TCCTCTGTGGGAGAATCAT (N)
Nc snR3	CGGGACTTCAGAAAGCCAT (N)
Nc snR10	CATAGCAGCATCAATCGCA (N)
Nc snR15	CATTAGAAGTTGTCTCCACG (N)
Nc snR18	TCTAATACAGAGGCTAACAC (N)
Nc snR20	AGACTGTTGAGCTTGATA (N)
Nc snR23	TTAGAACCAAGTCAACACCG (N)
Nc snR25	CGTGTCAGTCGCAACGGTTCT (N)
Nc snR30	AAACGCCAGACATAGCAGG (N)
Nc snR31	TCAGTTCGGGTGCCGTGTC (N)
Nc snR35	CCGCCAAAGCGCCCGAAGG (N)
Nc snR40	TCACTTCAAAGCCCAGCAA (N)
Nc snR42	ACTGTGACACTCTGCCCAAT (N+R)
Nc snR43	TGGTAGACCGCAGCGTTTGT (N+R)
Nc snR44	GCCTTGGACAGCCGTATTT (N+R)
Nc snR45	AACCCAACACAACATCGGCA (N+R)
Nc snR46A	GTGCTGTTGTGTTAATCATTTGCAG (N+R)
Nc snR46B	TAAATGCTGGTTTCGTCGTCA (N+R)
Nc snR47	GGTATTTAGCAACAGCATCGGC (N+R)
Nc snR48	TCAGATGGGAGCGAAAGA (N+R)
Nc snR49	TAGGTCGATATGGTGGAACG (N+R)
Nc snR50	ACCCACCCACTGCGGTAC (N+R)
Nc snR51	GTGATGTTGGTCTCATGGCC (N)
	GAGGGCTCTGAAGTAGCAAATA (R)
Nc ACA1	CATTGGTGACTCCAGTAACG (N+R)
Nc ACA2	TTCCTCTCGGCACGGACTAAA (N+R)
Nc ACA3	TTTGCGATGTTCGGCACTGTCT (N)
Nc ACA4	AAAGCCATACCCGAAAGCG (N)
Nc ACA5	AAGGGCTGTTTGTGGCATCA (N)
Nc ACA6	TTCTATCCTCCACTTTCCCC (N)
Nc ACA7	TCAGAATGAAACCACCGCCA (N)
Nc ACA8	GGGCAACGCATTCATAGCA (N)
Nc ACA9	CTCGGGTCATCCAAAGCATTCC (N)

Nc ACA10	AACTCCCTTCTCCCATCCGT (N)
Nc ACA11	AAGTGCGGTATTCCTCTGGG (N)
Nc ACA12	GGTGACCTGTCGGATGGAAA (N)
Nc ACA13	GCACCCAAGATTGTTCGCTG (N)
Nc ACA14	CGCACCAAGCGAACCTGAAC (N)
Nc ACA15	TAAAGGGAAGAGATGGAGAAGCA (N)
Nc ACA16	TCACGCCATCCAATCATTACAA (N)
Nc ACA17	CAGCACATCTTCGGCACAACCA (N)
Nc ACA18	GGGACGGGCAGACAAGATGATT (N)
Nc ACA19	ACGAGTCGGAACAACGAGCCTT (N)
Nc ACA20	AAGGCTGCGTTTGTTCTCGT (N+R)

C) The primers used for RT-PCR experiment of six box C/D snoRNA gene clusters were as follows.

Primer	Sequence (5’→3’)	Annotation
NC-C1R1	TGCTCGATGGGTCATCATAGTAA	(reverse transcription)
NC-C1R2	TCATAGTAATGATACCGGACAATGG	(reverse primer of PCR)
NC-C1F	CATCATCACCAAGCATCGCC	(forward primer of PCR)
NC-C2R1	GGGCATGTCGACTCTCCCACTAGAC	(reverse transcription)
NC-C2R2	CACTAGACGCAAGATCGTTTTCAG	(reverse primer of PCR)
NC-C2F	GGTCATGTCAACTGTCCAGGCA	(forward primer of PCR)
NC-C3R1	TAAAACTGAGAAATGTTAGCGTCCG	(reverse transcription)
NC-C3R2	AATGTTAGCGTCCGCCAACAAATCG	(reverse primer of PCR)
NC-C3F	GATGCTTCAGACTTCCTTCCACAG	(forward primer of PCR)
NC-C4R	CCCAGAGGTTGCCGAGTAT	(primer of reverse transcription and reverse primer of PCR)
NC-C4F	TCCTTCCCTCACCCTTCTAC	(forward primer of PCR)
NC-C5R	TCACTAAGGAGGGCAAAAGCAGAG	(primer of reverse transcription and reverse primer of PCR)
NC-C5F	GCTGGCAGGAACCGCTTCATTG	(forward primer of PCR)
NC-C5F2	AAATGTCTTCTTTTCCCAACTGA	(forward primer of PCR for co-transcription)
NC-C5R2	CGAGTGGAAACTGCGAATGTAG	(reverse primer of PCR for co-transcription)
NC-C6R1	TCCTCGGATCTACTCTCCACGCCC	(reverse transcription)
NC-C6R2	CCCGTGTCTTTCATTCCTTCA	(reverse primer of PCR)
NC-C6F	CGCGATACCGTCACAACAGA	(forward primer of PCR)
